# Lineage-specific symbionts mediate differential coral responses to thermal stress

**DOI:** 10.1186/s40168-023-01653-4

**Published:** 2023-09-26

**Authors:** Chenying Wang, Xinqing Zheng, Hagit Kvitt, Huaxia Sheng, Danye Sun, Gaofeng Niu, Dan Tchernov, Tuo Shi

**Affiliations:** 1 Key Laboratory of Marine Ecology Conservation and Restoration, Third Institute of Oceanography, Ministry of Natural Resources, Xiamen, 361005 China; 2 State Key Laboratory of Marine Environmental Science, College of Ocean and Earth Sciences, Xiamen University, Xiamen, 361102 China; 3 Observation and Research Station of Wetland Ecosystem in the Beibu Gulf, Ministry of Natural Resources, Beihai, 536015 China; 4 Marine Biology Department, The Leon H. Charney School of Marine Sciences, University of Haifa, 31905 Haifa, Israel; 5 Israel Oceanographic and Limnological Research, National Center for Mariculture, 88112 Eilat, Israel; 6 Marine Genomics and Biotechnology Program, Institute of Marine Science and Technology, Shandong University, Qingdao, 266237 China; 7 Southern Marine Science and Engineering Guangdong Laboratory (Zhuhai), Guangzhou, 510000 China

**Keywords:** Climate change, Coral microbiome, Coral reef, Symbiodiniaceae, Symbiont shuffling, Thermal resilience

## Abstract

**Background:**

Ocean warming is a leading cause of increasing episodes of coral bleaching, the dissociation between coral hosts and their dinoflagellate algal symbionts in the family Symbiodiniaceae. While the diversity and flexibility of Symbiodiniaceae is presumably responsible for variations in coral response to physical stressors such as elevated temperature, there is little data directly comparing physiological performance that accounts for symbiont identity associated with the same coral host species. Here, using *Pocillopora damicornis* harboring genotypically distinct Symbiodiniaceae strains, we examined the physiological responses of the coral holobiont and the dynamics of symbiont community change under thermal stress in a laboratory-controlled experiment.

**Results:**

We found that *P. damicornis* dominated with symbionts of metahaplotype D1-D4-D6 in the genus *Durusdinium* (i.e., PdD holobiont) was more robust to thermal stress than its counterpart with symbionts of metahaplotype C42-C1-C1b-C1c in the genus *Cladocopium* (i.e., PdC holobiont). Under ambient temperature, however, the thermally sensitive *Cladocopium* spp. exhibited higher photosynthetic efficiency and translocated more fixed carbon to the host, likely facilitating faster coral growth and calcification. Moreover, we observed a thermally induced increase in *Durusdinium* proportion in the PdC holobiont; however, this “symbiont shuffling” in the background was overwhelmed by the overall *Cladocopium* dominance, which coincided with faster coral bleaching and reduced calcification.

**Conclusions:**

These findings support that lineage-specific symbiont dominance is a driver of distinct coral responses to thermal stress. In addition, we found that “symbiont shuffling” may begin with stress-forced, subtle changes in the rare biosphere to eventually trade off growth for increased resilience. Furthermore, the flexibility in corals’ association with thermally tolerant symbiont lineages to adapt or acclimatize to future warming oceans should be viewed with conservative optimism as the current rate of environmental changes may outpace the evolutionary capabilities of corals.

Video Abstract

**Supplementary Information:**

The online version contains supplementary material available at 10.1186/s40168-023-01653-4.

## Background

Coral lives in symbiosis with a plethora of interwoven microorganisms, including bacteria, archaea, fungi, and microalgae, which are known to enhance the ability of corals to synthesize calcium carbonate skeletons [1]. Photosynthetic dinoflagellates in the family Symbiodiniaceae [2, 3] are algal symbionts of many cnidarians including corals, sea anemones, jellyfish, and hydras. Nutrient exchanges between scleractinian corals and Symbiodiniaceae underpin the success of reef-building corals as habitat engineers in coral reef ecosystems [4]. Reef corals are reliant on the translocation of Symbiodiniaceae-derived compounds in support of calcification demands, and in exchange, Symbiodiniaceae receive metabolic byproducts required for growth and photosynthesis [5]. This cnidarian–algal association is of particular importance to coral reefs and plays a key role in carbon sequestration in the context of global climate change [6, 7], as elevated seawater temperatures are threatening coral reef ecosystems worldwide, causing more frequent and severe mass coral “bleaching” (the loss or expulsion of algae from the host corals) [8].

It is generally suggested that symbiont community structure and functional diversity shape the energy balance and stress tolerance of host corals, which are important factors in sustaining their symbiotic relationship under thermal stress [9–11]. However, the specific impacts of symbiont composition and abundance on host corals’ fitness and ecological success remain poorly resolved [10, 12, 13]. For example, while the role of Symbiodiniaceae in fueling host metabolism with nutrient and energy supply has long been recognized [5], only recently has the divergent capacity of algal symbionts in translocating photosynthates and regulating host trophic plasticity been examined [14–16]. Moreover, studies relating algal symbiont genotypes to thermal stress resilience of reef corals are rather limited [13, 14].

The extent to which corals adapt to a changing climate relies in part on the genetic variation of Symbiodiniaceae [17, 18] and the environmental conditions under which the coral–dinoflagellate mutualisms develop [19, 20]. Different Symbiodiniaceae strains or species are likely to differ in their intrinsic adaptive capacity, and stress-tolerant traits may evolve in some opportunistic symbiont variants [21]. Numerous studies have confirmed that corals harboring symbiont strains in the genus *Durusdinium* (formerly clade D) show greater thermal tolerance than corals hosting symbionts belonging to the genus *Cladocopium* (formerly clade C) [22–24]. As a result, thermally tolerant *Durusdinium* strains are often found to be prevalent in reefs that have survived episodes of severe mass bleaching or exposed to long-term stressed conditions [25]. The symbiont “switching” (i.e., acquirement of new, thermally resistant Symbiodiniaceae genera) or “shuffling” (i.e., *in hospite* proliferation of stress-tolerant symbionts, usually present at low to undetectable levels prior to bleaching) hypotheses have generally been accepted to explain increased coral resilience to thermal stress [9, 26]. However, recent evidence revealed that symbiont shuffling comes at a physiological cost to the cnidarian host, and conspecific corals may acquire less photosynthates from symbionts in the genus *Durusdinium* than from symbionts in other genera, leading to significant reduction of calcification rates [27–29]. In this context, the trade-offs in the flow of energy and matter in coral–algal symbiosis are directly related to holobiont function in reef ecosystems [30]. However, the underlying ecological benefit and long-term stability of such adaptive changes are unclear [31], and there remains a high degree of uncertainty over how these trade-offs are mediated by thermal stress [29].

Notably, symbiont recombination across broad cladal boundaries may not be suitable to describe all the differences in corals’ bleaching susceptibility [32]. Colonies of several coral species exhibit no changes in their symbiont communities during exposure to variable temperatures or bleaching stress [33, 34]. Moreover, differential bleaching susceptibility has been observed in corals harboring thermally sensitive versus resistant symbionts within *Cladocopium* rather than symbiont switching/shuffling [35, 36]. In the Persian Gulf, the world’s hottest sea with extremely high seasonal temperatures (up to 35°C), *Cladocopium thermophilum* of C3 type is the year-round prevalent symbiont whereas *Durusdinium* is essentially absent [37, 38]. These findings suggest that, in addition to the thermally tolerant cosmopolitan symbiont generalist such as *Durusdinium trenchii* of D1a/D1-4 type [31, 39], divergence in the physiology of opportunistic symbiont specialists is an equivalently important factor for increased coral resilience [31, 36, 38]. These contradictory observations have been linked to evolutionary differences among host and symbiont species in their capacity to adapt and/or acclimate to heat stress [35, 36]. Alternatively, most corals tend not to change their dominant symbionts to a different genus, unless they naturally host multiple Symbiodiniaceae genera or the warming levels they experienced have been strong enough to drive symbiont community changes [40]. Despite the notion that the vast majority of corals seem to associate with one dominant algal symbiont, the functional importance of rare background symbionts remains to be determined [41].

Acclimatization of corals to thermal stress involves symbiont physiological plasticity at both inter- and intra-genus levels, hence defining and measuring symbiont plasticity requires simultaneous assessment of different symbiont populations while characterizing physiology of each specific symbiont type. However, previous studies investigating symbiotic plasticity used either isolated Symbiodiniaceae strains [16, 42], or symbionts within distinct coral species [15, 43], but rarely compared physiological performance directly between different types of symbionts within the same coral species [9, 44]. Meanwhile, the relevance of this adaptive response is equivocal owing to conflicting reports of symbiont fidelity and flexibility mentioned above [33, 41]. In this study, we first investigated the dominant symbiont types of the widely distributed coral *Pocillopora damicornis* in the southern Hainan Island, China. Corals in this region live in a stressful local environment due to increased human activities [45], which can thus serve as a study site to understand the fate of coral–algal associations under future global warming scenarios. Secondly, we tested the symbiont shuffling hypothesis by manipulating conspecific *Pocillopora damicornis* species dominated with distinct Symbiodiniaceae strains (thermally sensitive metahaplotype C42-C1-C1b-C1c vs. thermally tolerant D1-D4-D6) in a laboratory-controlled thermal stress experiment. By exploring physiological responses of both the coral host and symbiont, combined with isotopic tracing of photosynthetically fixed carbon and quantification of symbiont community change, we aimed to investigate the role of distinct symbiont types in mediating coral responses to elevated temperatures with potential implications for the mechanistic basis of bleaching resistance.

## Materials and methods

### Coral collection and maintenance

Colonies of *P. damicornis* were collected by SCUBA diving at water depths of 3–5 m from two fringing reefs, Luhuitou (LHT; 18°12′7′′N, 109°28′5′′E) and Houhai (HH; 18°16′40′′N, 109°44′3′′E), located at the southern tip of Hainan Island in the South China Sea (Supplementary Fig. S1, Tables S1 and S2). Colony replicates were generally separated by 2–3 m across each reef. The collected corals were transferred to the indoor husbandry facility at the Third Institute of Oceanography, Ministry of Natural Resources, China, and cultivated in an aquarium with 1000 L of recirculated artificial seawater (ASW) at a temperature of 26°C and photosynthetically active radiation of 150 μmol photons/m^2^/s provided by metal halide lamps (Phillips, Amsterdam, Netherlands) over a 12-h/12-h light/dark cycle. To minimize perturbations from environmental sampling, the coral colonies were grown in the aquarium for 6 months, followed by fragmentation and further acclimation for two more months prior to the experimental manipulation. No changes in dominant symbiont genotype were found during the coral maintenance [10].

### Experimental setup of thermal stress simulation

**Fig. 1:**
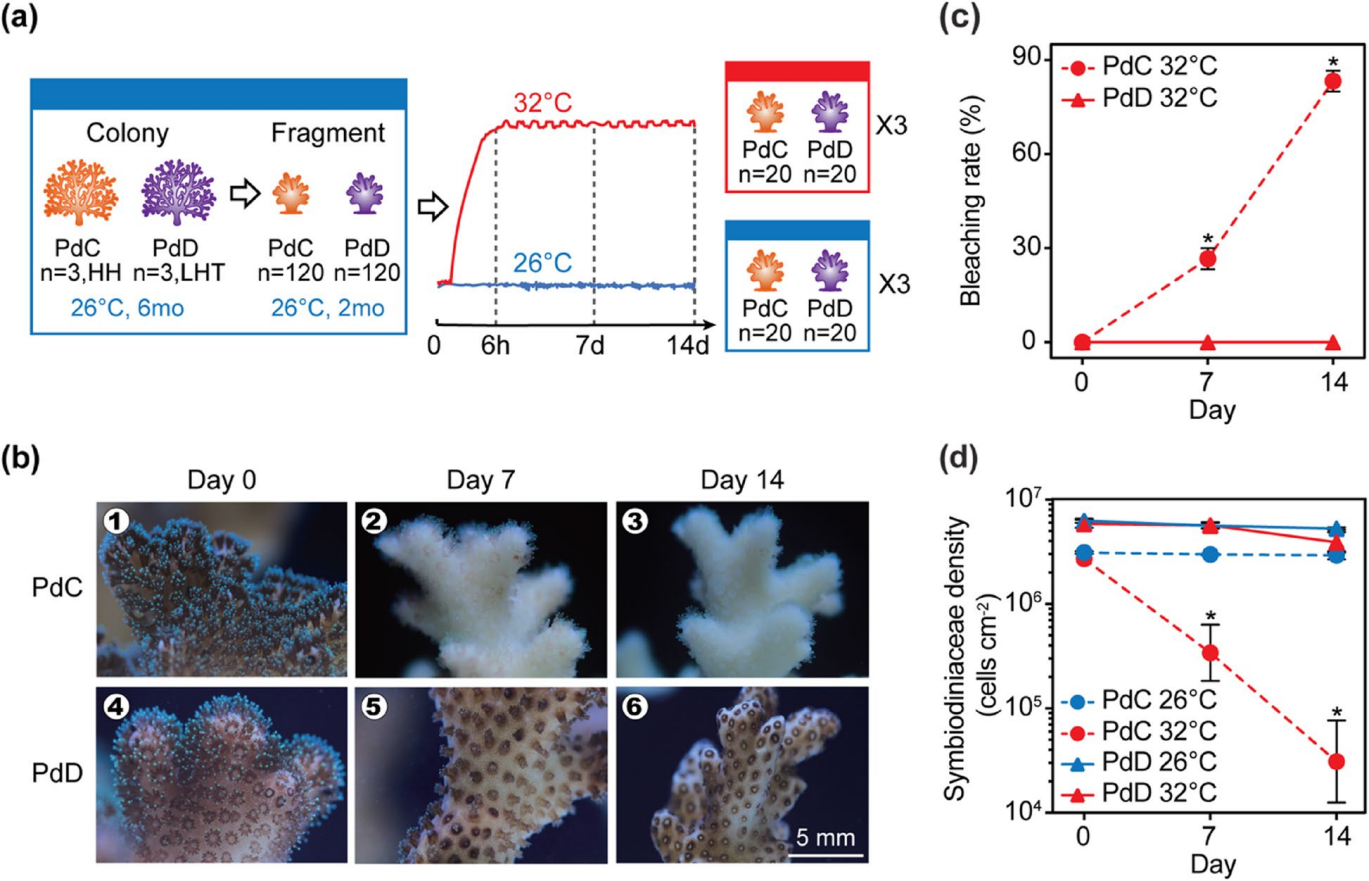
Experimental setup and bleaching susceptibility of *P. damicornis* harboring distinct genera of Symbiodiniaceae. **a** Schematic diagram showing the maintenance, fragmentation, and manipulation of *P. damicornis* harboring *Cladocopium* (PdC) or *Durusdinium* (PdD) under control (26°C, blue) or heated (32°C, red) conditions. Sampling time points are indicated with dashed vertical lines. **b** Morphological changes of PdC (1–3) or PdD (4–6) at different stages of the thermal stress. **c** Bleaching rates of PdC or PdD at different stages of the thermal stress. **d** Areal Symbiodiniaceae density of PdC or PdD at control (blue) or elevated temperature (red). Values are means ± standard error (*n* = 6). Asterisks (*) indicate significant difference between control and thermal stress at designated time points (Tukey’s post hoc test, *p* < 0.05)

Colonies of *P. damicornis* dominated with symbionts in the genus *Cladocopium* (hereafter PdC) or *Durusdinium* (hereafter PdD) were identified (see details below for Symbiodiniaceae genotyping) and chosen for the thermal stress simulation experiment. Triplicate distinct colonies of PdC (*n* = 3 from HH) and PdD (*n* = 3 from LHT) were each cut into 40 ramets of ~ 2–3 cm long, resulting in a total of 120 fragments per symbiont type. For each parental colony, the 40 ramets were distributed into six 80-L tanks (each containing 6–7 ramets of the same colony), three of which were maintained at control temperature of 26°C and the other three were heated to 32°C with a gradual increase of 1°C per hour. The heat stress was applied for 14 consecutive days, with water temperatures recorded using a HOBO temperature logger (Onset Corp., Bourne, MA, USA) (Fig. 1a). The coral fragments were fate-tracked throughout the entire experiment. Subsets of the fragments were used for measurements of symbiont photochemistry and coral calcification, Symbiodiniaceae count and symbiont community structure assessment, as well as the uptake of stable carbon isotope (^13^C).

### Host and symbiont genotyping

Genomic DNA of the coral holobiont was extracted with the cetyltrimethylammonium bromide (CTAB) method [46] using coral tissue stripped from the skeletons of ~ 1 cm^2^ via vortex in DNA lysis buffer (10 mM Tris–HCl, pH 8.0; 100 mM EDTA, pH 8.0; 0.5% [w/v] SDS). Coral species was initially identified based on morphological traits of skeleton as discerned in the scanning electron microscopy [47] and then verified with PCR amplifying the full-length internal transcribed spacer (ITS) region (i.e., ITS1-5.8S-ITS2, approximately 1300 bp) of coral nuclear ribosomal DNA (nrDNA) using Anthozoa-specific primers [48]. The coral sequences were aligned against reference sequences for *Pocillopora* and closely related taxa from NCBI. For coral identity, PCR amplicons from all the colonies matched with reference sequences for *P. damicornis* and hence confirmed their initial identification based on morphology (Supplementary Figs. S2 and S3).

Symbiont genotypes were determined by PCR amplifying the ITS region 2 (ITS2) of Symbiodiniaceae nrDNA utilizing primers “ITS2-Dino” and “ITS2-rev2” [49]. PCR amplicons were gel-purified, cloned, and then Sanger-sequenced. At least six fragments (~ 2–3 cm) per colony of at least three colonies of PdC or PdD on each reef were assayed prior to and at the end of the temperature manipulations. For each fragment, 6–12 clones of the PCR amplicons were Sanger-sequenced to verify the coral species and the dominant symbiont types. The symbiont sequences were compared to a custom Symbiodiniaceae ITS2 database unifying the Arif et al. [50] and Cunning et al. [51] data sets [52]. Phylogenetic trees for coral ITS and symbiont ITS2 were reconstructed using PAUP* software (v4.0a build 165) with the HKY + G substitution model estimated by MrModelTest [53] based on alignments generated with MAFFT v7.427 software [54]. Haplotype networks for symbiont ITS2 were constructed using the program HapStar [55] with a minimum spanning matrix of absolute pairwise nucleotide difference determined by haplotypes package v1.1.2 in R (https://www.r-project.org/). For symbiont identity, PCR amplicons from all the colonies matched with *Cladocopium* spp. or *Durusdinium* spp., with varying *Durusdinium* to *Cladocopium* ratios (Supplementary Figs. S4 and S5).

### Determination of Symbiodiniaceae density

Duplicate ramets per colony (*n* = 6, from two of the three treatment tanks) were preserved at –80°C for Symbiodiniaceae count on a weekly interval (day = 0, 7, 14). Coral tissue was removed from the skeleton using an airbrush connected to a reservoir of 0.22-μm pore-size filtered ASW, and the skeletons were used for surface area measurement afterwards. The tissue was homogenized and the homogenate was divided into aliquots for Symbiodiniaceae count (fixed in Lugol’s solution) and symbiont community structure assessment (stored in DNA lysis buffer). Symbiodiniaceae cells were counted in triplicates for a total of 36 fragments (one PdC and one PdD fragment per tank at 3 time points) using a hemocytometer (Boeco, Hamburg, Germany) under a light microscope (Nikon, Tokyo, Japan). The Symbiodiniaceae cell counts were normalized to the estimated surface area of individual *P. damicornis* fragments as previously described [56]. Symbiodiniaceae density was determined for both healthy and bleached corals. The rates of coral bleaching (occurred only in PdC at 32°C) were calculated as the percentage of bleached fragments in all the remaining fragments at designated time points. Coral bleaching is generally checked with the color card or RGB methods [57], during which corals are repeatedly taken out of the tank. To minimize this stressful manipulation, we arbitrarily assessed coral bleaching by comparing heated and control corals, and corals were considered bleached only when the entire fragment was visibly paled.

### Measurement of symbiont photosynthetic efficiency

Photosynthetic efficiency of algal symbiont was assessed with quantum yield of chlorophyll *a* fluorescence measured with an underwater diving-PAM fluorometer (Walz, Effeltrich, Germany), with parameters set as follows: measuring light intensity = 8, signal damping = 2, gain = 6, saturating light pulse intensity = 8, saturating light pulse width = 1, actinic light intensity = 4, and actinic light width = 0:30. Measurements of the maximum (*F*_*v*_*/F*_*m*_) and effective ($\Delta$*F/F*_*m*_*'*) quantum yield of photosystem II (PSII) were conducted every day upon 2 h into the dark and light period, respectively. The maximum excitation pressure over PSII (*Q*_*m*_) was calculated according to Iglesias-Prieto et al. [58]:

1 $${\text{Q}}_{\text{m}} \, \text{=} \, {1}- [(\Delta {\text{F}}/{\text{F}}_{\text{m}}^{\prime} )/( {\text{F}}_{\text{v}}/{\text{F}}_{\text{m}}) ],$$

where *Q*_*m*_ is a normalized metric. To isolate the effect of thermal stress, the *Q*_*m*_ in the control treatment was subtracted from the corresponding *Q*_*m*_ in the thermal stress to calculate Δ*Q*_*m*_. Thus, Δ*Q*_*m*_ is comparable among different photosymbionts and reflects the relative degree to which they adjust PSII to the thermal stress. Triplicate ramets per colony (*n* = 9, one per treatment tank) were taken to measure symbiont photochemistry (twice a day). All measurements were conducted in triplicates per coral fragment in order to minimize variations.

### Measurement of coral calcification rates

The same subset of coral fragments used for photosynthetic measurements were also used to calculate coral calcification rates (*n* = 9), but on a weekly interval (day = 0, 7, 14). Coral calcification rates were determined with the buoyant weight (BW) technique measuring the increase in skeletal mass as previously described by Davies [59]. Briefly, coral ramets were suspended in a temperature-controlled seawater bath from 0.05-mm-diameter tungsten wires attached to the underside of an analytical balance that weighs to an accuracy of 0.0001 g. The balance was mounted on a weighing chamber sealed with transparent acrylic panels. Optimal weighing conditions were obtained when the weighing apparatus was located in a room with stable air temperature close to that of the seawater. The daily skeletal mass increase was normalized to initial skeletal surface area (*A*_initial_) with the equation:

2 $${\mathrm{Calcification}\;\mathrm{rate}}=\;\frac{{\mathrm{BW}}_{\mathrm{final}}-{\mathrm{BW}}_{\mathrm{initial}}}{{\mathrm A}_{\mathrm{initial}}}\times\frac{1}{\mathrm{day}}\times100\%$$

### Isotopic tracing of photosynthetically fixed carbon

At the end of the thermal stress simulation experiment (day = 14), carbon isotopic tracing was performed in each control (26°C) and heated (32°C) tank with coral fragments suspended in duplicated 1-L polycarbonate bottles filled with recirculated ASW from the same tank (*n* = 6, one fragment per bottle). A total of 24 coral fragments from 3 PdC and 3 PdD parental colonies (4 fragments per colony) were evenly distributed among the 6 tanks. ^13^C-labeled sodium bicarbonate (99 atom% ^13^C; Cambridge Isotope Laboratories, Andover, MA, USA) was added into each bottle at a final tracer concentration of 70 μmol L^−1^. The bottles were incubated for 24 h. Samples were harvested at the end of the incubation, followed by immediate snap-freezing in liquid nitrogen and storage at –80°C until further processing. Meanwhile, one unlabeled PdC or PdD fragment was also taken from each tank as controls to assess the natural isotope ratios. Coral tissue was removed from the skeleton by an airbrush connected to a reservoir of 0.22-μm pore-size filtered ASW. The slurry was homogenized and the host and symbiont fractions were separated by centrifugation and collected on precombusted GF/F filters following a previously described protocol [60]. Carbon stable isotopic values and organic carbon content for calculating total carbon biomass of coral holobiont were determined using a Delta V Plus isotope ratio mass spectrometry (IRMS) (Thermo Fisher Corporation, Waltham, MA, USA) interfaced with a Flash HT 2000 elemental analyzer (EA) (Thermo Fisher Corporation, Waltham, MA, USA). The rates of ^13^C enrichment were calculated as per Hama et al. [61]. The reproducibility for δ^13^C measurement was better than 0.3‰. Samples with erroneous isotopic values were discarded and only those with enriched ^13^C isotopic ratios higher than the natural carbon isotopic fractionation were considered valid. The uptake rates of carbon tracer (^13^C) in the enriched fractions (*C*_tracer_) have all been normalized to total organic carbon biomass (*C*_tissue_) to allow direct comparison between corals and Symbiodiniaceae.

### Characterization of symbiont community structure

To screen the relative *Durusdinium* to *Cladocopium* abundance of the symbionts, quantitative PCR (qPCR) was performed with *Cladocopium-* or *Durusdinium*-specific primers targeting the Symbiodiniaceae nrDNA 28S region [62] using biological hexaplicates (*n* = 6) of coral fragments harvested prior to the experiment (pre-treatment), as well as those harvested at designated time points (day = 0, 7, 14) during the thermal stress manipulation (control and heated). The qPCR reactions were run in technical triplicates on a CFX96 Real Time System (Bio-Rad Laboratories, Hercules, CA, USA) using iTaq Universal SYBR Green Supermix (Bio-Rad Laboratories, Hercules, CA, USA) according to the manufacturer’s instructions. The ratio between *Durusdinium* and *Cladocopium* cells was calculated using the formula:

3 $${\text{D}}/{\text{C}}\text{ } = { 2}^{-\Delta {\text{C}}_{\text{T}}} \, = {2}^{-\left[{\text{C}}_{\text{T (Primer D)}}-{\text{C}}_{\text{T (Primer C)}}\right]},$$

where *C*_*T* (Primer C)_ and *C*_*T* (Primer D)_ are the threshold cycle (*C*_*T*_) specific to the *Cladocopium* and *Durusdinium* reaction, respectively [62].

To quantify the degree of symbiont shuffling, a symbiont shuffling index was derived given *Durusdinium* proportion normalized to its minimum (–1, i.e., complete dominance by *Cladocopium*) to maximum (+ 1, i.e., complete dominance by *Durusdinium*) values based on the symbiont relative abundance. This index is similar to the symbiont shuffling metric proposed by Cunning et al. [12]. The symbiont community was also assessed using all the cloned ITS2 sequences that matched reference sequences of known Symbiodiniaceae types [52] based on BLAST (Supplementary Figs. S4 and S5). The observed dominant Symbiodiniaceae types were assembled and scored given the number of their matched clones (i.e., abundance) in each sample. The resulting assignment scores were assimilated into a matrix of symbiont metahaplotypes, which was analyzed in a similar fashion to DGGE band presence/absence profiling for symbiont community composition [63]. As no ITS2 types were in common between *Cladocopium*- and *Durusdinium*-dominated *P. damicornis*, a “dummy species” was added to the original abundance matrix, with value 1 for all samples, to counter for the effect of sparse samples. A non-metric multidimensional scaling (NMDS) plot was then constructed using this zero-adjusted Bray–Curtis dissimilarity [64].

### Statistical analyses

To determine whether the short-term heat stress acclimation affects coral growth, a generalized linear mixed-effects model was employed to compare the effects of fixed (temperature, time, and genotype) and random (tank and colony) factors [65] on the following physiological traits: Symbiodiniaceae density, *F*_*v*_/*F*_*m*_, Δ*F*/*F*_*m*_*'*, calcification rates and symbiont relative abundance. The Shapiro–Wilk test and the Levene’s test were conducted to verify assumptions of normality and homogeneity of variances. One-way analysis of variance (ANOVA) was performed to compare coral bleaching rate, Symbiodiniaceae density, symbiont photochemistry (*F*_*v*_/*F*_*m*_ and Δ*F*/*F*_*m*_*'*) and calcification rate between samples exposed to different thermal stress time, followed by the post hoc Tukey’s multiple comparison test when the differences were significant (*p* < 0.05). Three-way ANOVA was performed to compare the impacts of temperature, time, and symbiont genotype on Symbiodiniaceae density, followed by the Kruskal–Wallis test as the data violated the ANOVA assumptions. Linear regressions were conducted to model symbiont adaptation to thermal stress (i.e., Δ*Q*_*m*_), changes in symbiont relative abundance (*Durusdinium* to *Cladocopium* ratio) over time, as well as the effects of thermal treatment on translocation of photosynthetically fixed carbon to the hosts by different genera of symbiont. Principal component analysis (PCA) was conducted to compare the effects of multiple physiological traits (*F*_*v*_*/F*_*m*_, Δ*F/F*_*m*_*'*, Symbiodiniaceae density, calcification rate, carbon fixation, carbon translocation, and symbiont relative abundance) on the overall host response to heat stress. All statistical analyses were performed with R version 4.0.3 [66].

## Results

### Bleaching susceptibility of *P. damicornis* harboring distinct symbionts

The conspecific colonies of *P. damicornis* harboring genetically distinct Symbiodiniaceae strains exhibited differential bleaching susceptibility upon thermal stress. *P. damicornis* dominated by metahaplotype C42-C1-C1b-C1c in the genus *Cladocopium* (Supplementary Fig. S4) started to bleach after 7 days of thermal stress (Fig. 1b), with the bleaching rate increasing from 26.7% on day 7 to 83.3% on day 14 (Fig. 1c, Supplementary Table S3). In contrast, *P. damicornis* dominated by metahaplotype D1-D4-D6 in the genus *Durusdinium* (Supplementary Fig. S5) showed no sign of bleaching even after 14 days of thermal stress (Tukey’s test,* p* > 0.05) (Fig. 1c), except that the coral polyps appeared shrunken and condensed (Fig. 1b). The Symbiodiniaceae cell density in PdC declined by 98% after 14 days of thermal stress, but that in PdD did not change significantly between the control and thermal stress (Tukey’s test,* p* > 0.05) (Fig. 1d, Supplementary Tables S3, S4 and S5). Noticeably, under control temperature, Symbiodiniaceae cell density was consistently higher in PdD than in PdC (Fig. 1d, Supplementary Tables S4 and S6).

### Lineage-specific symbiont photochemical responses to thermal stress

**Fig. 2:**
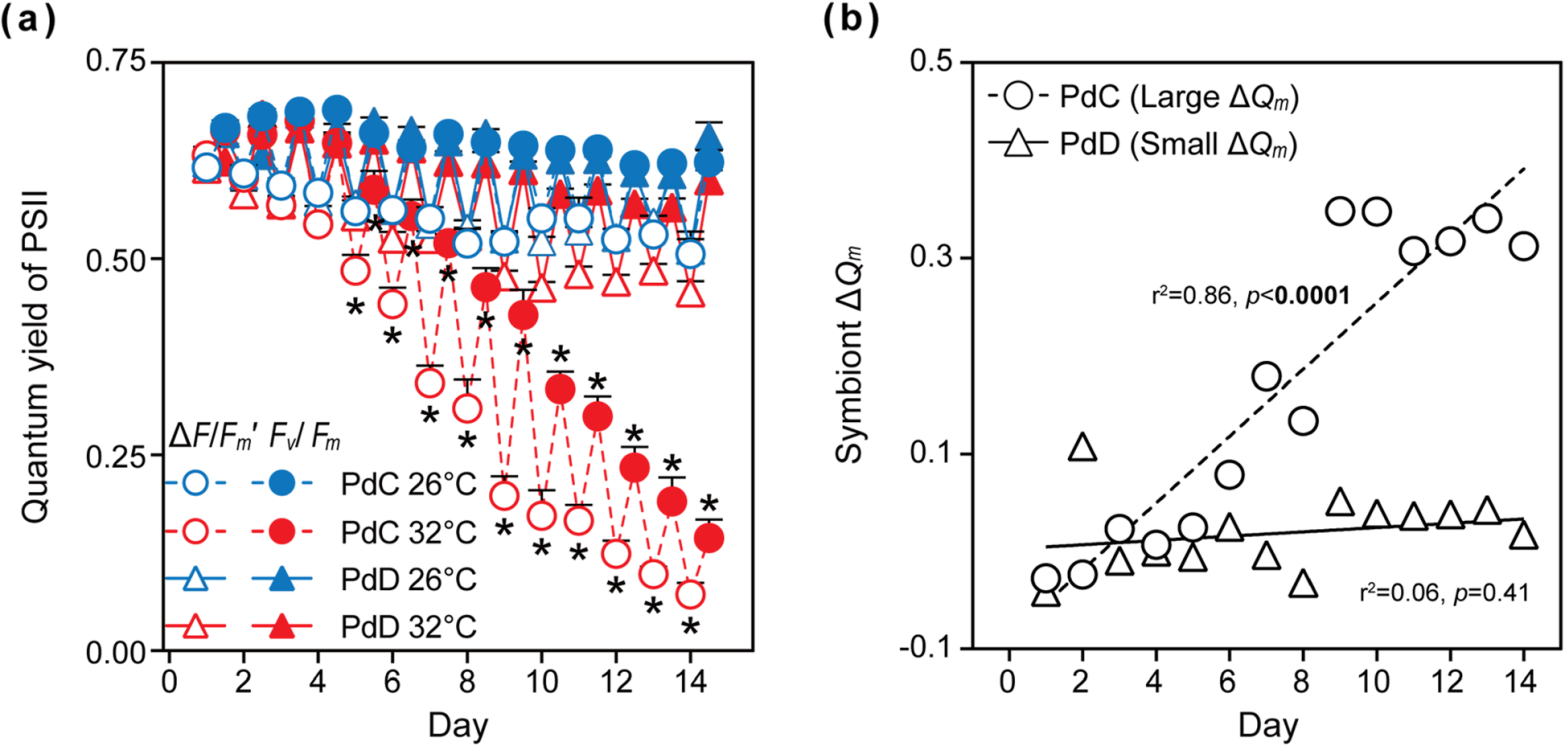
Photosynthetic efficiency of *P. damicornis* subjected to thermal stress. **a** Diurnal oscillations in the maximum (*F*_*v*_/*F*_*m*_) and effective (∆*F/F*_*m*_*'*) quantum yield of PSII charge separation. **b** Changes in PSII adjustment to thermal stress (∆*Q*_*m*_). Values are means ± standard error (*n* = 9). Asterisks (*) indicate significant difference between control and thermal stress at designated time points (Tukey’s post hoc test, *p* < 0.05)

Starting on day 5, photosynthetic efficiency of the symbionts in PdC was negatively affected by the thermal stress (Tukey's test, *p* < 0.05) (Fig. 2a). The maximum quantum yield of photosystem II (PSII) in the heated group culminated in a sharp 77% decrease on day 14 relative to the control group (Fig. 2a, Supplementary Table S3). In contrast, no significant reduction in the symbionts’ PSII quantum yield was observed in PdD under the thermal stress (Tukey’s test, *p* > 0.05) (Fig. 2a). The PSII adjustment to thermal stress (∆*Q*_m_) was large in PdC (0.17 ± 0.04, Tukey’s test, *p* < 0.05) but small in PdD (0.02 ± 0.01, Tukey’s test, *p* > 0.05) (Fig. 2b). A small Δ*Q*_m_ for PdD suggests the greater capacity for the D1-D4-D6 symbiont to engage photorepair mechanisms or effectively resist photodamage.

### Coral calcification rates, symbiotic carbon fixation, and translocation

**Fig. 3:**
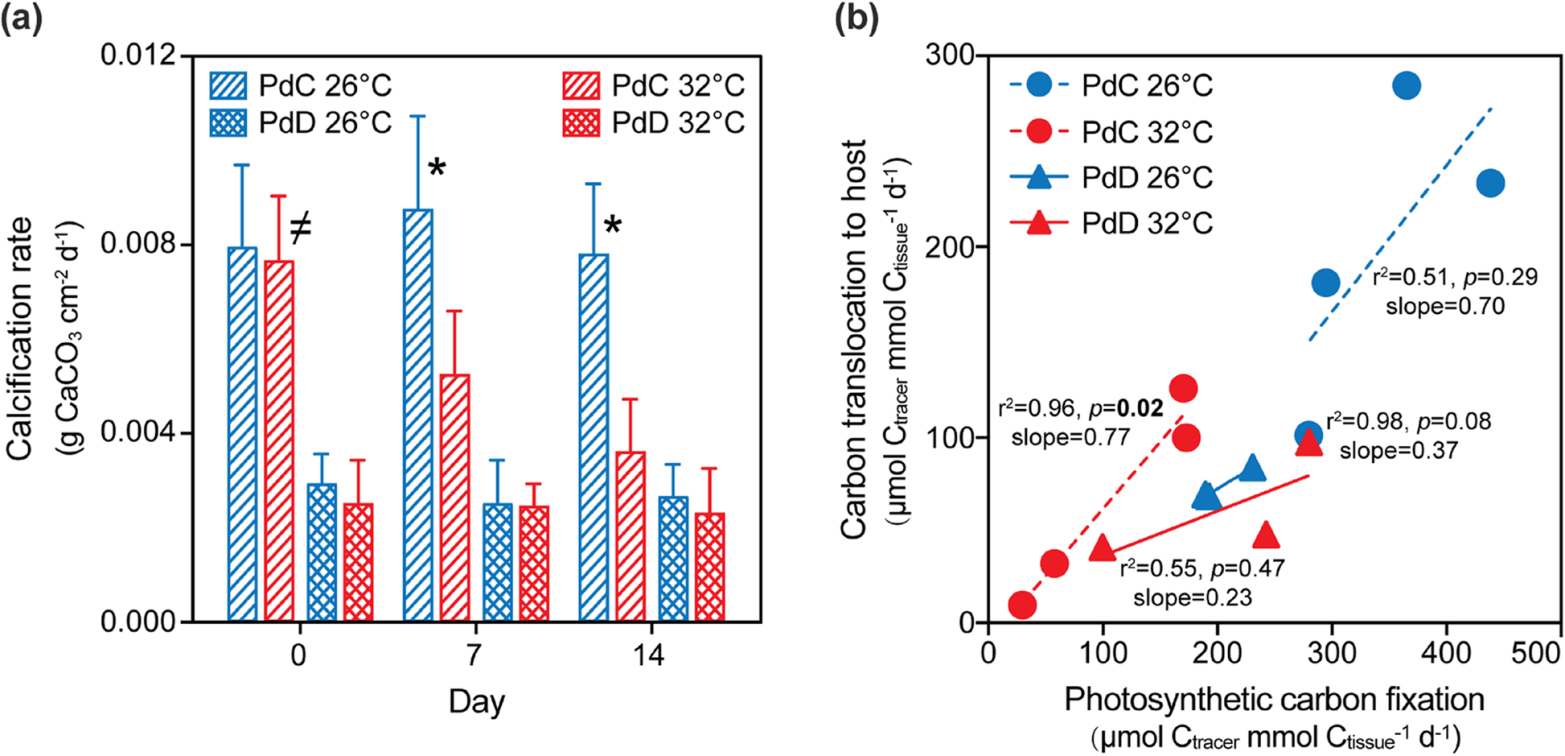
Calcification rates and translocation of photosynthetically fixed carbon in *P. damicornis*. **a** Calcification rates expressed as daily skeletal mass increments normalized to skeletal surface area. **b** Translocation of photosynthetically fixed carbon from symbionts to the host at control and elevated temperatures, as determined with stable isotopic tracer analysis. Values for calcification are means ± standard error (*n* = 9). Asterisks (*) indicate significant difference between control and heat stressed PdC at designated time points (Tukey’s post hoc test, *p* < 0.05). The inequality sign ( ≠) indicates significant difference between corals harboring *Cladocopium* or *Durusdinium* at the beginning of the thermal stress manipulation (Tukey’s post hoc test, *p* < 0.05)

At control temperature, the coral calcification rate in PdC was about three times higher than that in PdD. Under elevated temperature, the calcification rate in PdC decreased by 40% on day 7 and 50% on day 14 as compared to control (Tukey’s test, *p* < 0.05, Supplementary Table S3), whereas that in PdD showed no difference between the two temperatures (Tukey’s test,* p* > 0.05) (Fig. 3a). At control temperature, the rates of both photosynthetic carbon fixation and subsequent translocation of photosynthetically fixed carbon (PFC) to the coral host were higher in PdC than in PdD. Moreover, the partitioning of PFC to the host was significantly more efficient in PdC than in PdD, as indicated by the different slopes of the two trend lines (Fig. 3b, Supplementary Fig. S6). While this PFC partitioning efficiency remained constant per each holobiont under the control and elevated temperatures (i.e., no change in the slopes of the trend lines between the two temperatures) for both PdC and PdD (Supplementary Fig. S6), the rates of carbon fixation and translocation significantly slowed down in PdC after 14 days of thermal stress (Tukey’s test, *p* < 0.05), but appeared unaffected in PdD (Tukey’s test,* p* > 0.05) (Fig. 3b).

### Symbiont dominance sustained regardless of thermal stress

**Fig. 4:**
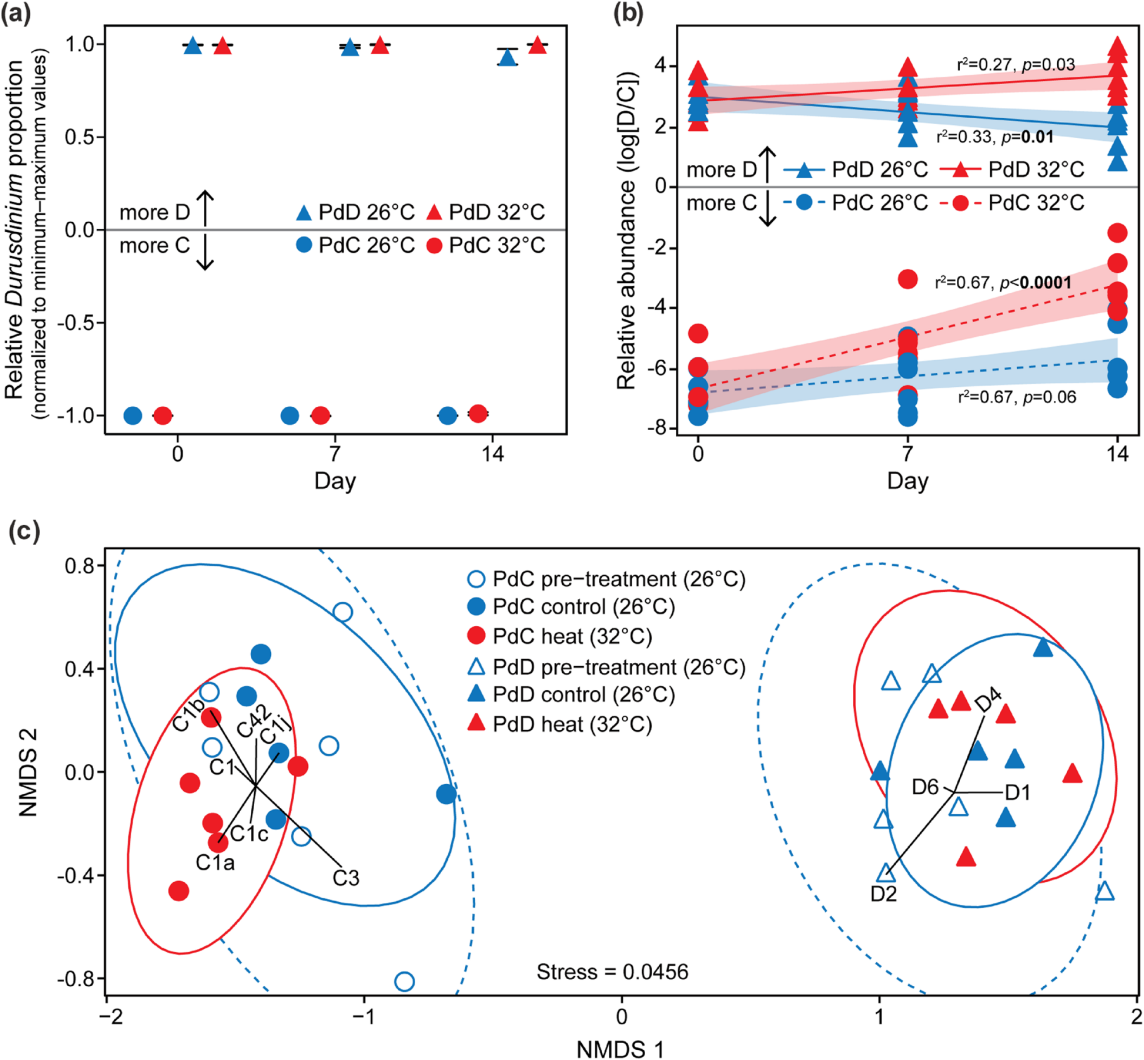
Symbiont community structure prior to, during, and at the end of the temperature manipulations. **a** Lineage-specific symbiont dominance throughout the experimentation period. Relative proportions of *Durusdinium* symbionts at designated time points of the temperature manipulation are normalized to a range of –1 (complete dominance by *Cladocopium*) to + 1 (complete dominance by *Durusdinium*) to infer the degree of symbiont shuffling. Values are means ± standard error (*n* = 6). Standard error was 0 for PdC at 26°C both on Day 0 and Day 7. **b** Changes in relative abundance of different genera of symbionts, expressed as log_10_[D/C] by calculating *Durusdinium* to *Cladocopium* ratio. Line and shaded area represent logarithmic regression and 95% confidence interval, respectively. **c** Non-metric multidimensional scaling (NMDS) ordination of symbiont communities constructed based on a Bray–Curtis dissimilarity matrix, given the relative abundance of dominant symbiont types detected during the aquarium maintenance (open dots/triangles) and at the end of temperature manipulation (solid dots/triangles). Grouping based on complete linkage at 90% similarity is indicated by circles for each treatment (dashed, pre-treatment 26°C; solid blue, control 26°C; solid red, heat 32°C). D *Durusdinium,* C *Cladocopium*

To test potential in-tank “shuffling” of algal symbionts, the overall *Durusdinium* to *Cladocopium* ratio was used as a proxy of relative abundance of the two symbiont types and was monitored based on qPCR over the course of the experiment. Both PdC and PdD sustained their initial symbiont dominance (> 99% of the symbiont community) regardless of the temperature difference (Fig. 4a). However, the sensitive qPCR assay revealed subtle changes in the relative abundance of the *Durusdinium* and *Cladocopium* symbionts, suggesting a “symbiont shuffling” in the background rare biosphere (Fig. 4b). Under thermal stress, the relative *Durusdinium* to *Cladocopium* ratio increased over time in both holobionts, but it increased much faster in PdC than in PdD (Tukey’s test, *p* < 0.05). Under control temperature, the ratio increased, albeit not significantly, in PdC (Tukey’s test, *p* > 0.05), but decreased significantly in PdD (Tukeys test, *p* < 0.05) (Fig. 4b).

The symbiont community composition was further assessed based on the cloned and annotated Symbiodiniaceae ITS2 sequences, by characterizing the dominant symbionts prior to and at the end of the temperature manipulations (pre-treatment and control at 26°C and heated at 32°C). Similar to the qPCR results, the symbiont community composition remained unchanged in both PdC and PdD throughout the experimentation period (Fig. 4c). The symbiont community was primarily comprised of closely related taxa (1–3 nucleotide difference in their ITS2 sequences) belonging to the same genus, suggesting no obvious shuffling across different genera. The PdD was associated with the stress-tolerant symbionts of metahaplotype D1-D4-D6 [67], whereas the PdC was dominated with stress-sensitive symbionts of metahaplotype C42-C1-C1b-C1c [63] (Supplementary Figs. S4 and S5).

## Discussion

### Coral thermal susceptibility is correlated with intrinsic symbiont photophysiology

In this study, by subjecting the same coral species hosting genetically distinct symbionts to increased temperature, we were able to correlate the physiological response of both partners to thermal stress with no significant tank and colony effects (Supplementary Fig. S7 and Table S4). After 2 weeks of thermal stress, *P. damicornis* hosting symbionts of metahaplotype C42-C1-C1b-C1c in the genus *Cladocopium* (i.e., PdC holobiont) bleached with decreased rate of calcification, whereas its counterpart hosting symbionts of metahaplotype D1-D4-D6 in the genus *Durusdinium* (PdD holobiont) showed no signs of bleaching or compromised calcification (Figs. 1b and 3a). Concomitantly, the *Cladocopium* in PdC exhibited significant decrease in photosynthetic efficiency and translocation of the PFC to the coral host under thermal stress. In contrast, photochemistry of the *Durusdinium* in PdD was barely affected, resulting in almost identical rates of PFC translocation to the coral host between the control and thermal stress (Figs. 2a and 3b). In addition, the higher cell density of *Durusdinium* naturally harbored by PdD could be a way of ameliorating *Durusdinium* inefficiency in supplying PFC under control temperature, which is consistent with previous findings that the PFC was negatively correlated with the Symbiodiniaceae density (Fig. 1d) [35, 68–70]. On the other hand, the smaller cell size of *Durusdinium* relative to *Cladocopium* [2, 71] may contribute to density stability due to the limited symbiosome space, facilitating PFC translocation to the coral host under thermal stress in PdD (Fig. 3, Supplementary Fig. S7) and thus favoring holobiont fitness.

Coral bleaching caused by high temperature has frequently been attributed to photoinhibition of photosynthetic electron transport, which leads to subsequent photodamage to PSII and production of reactive oxygen species (ROS) [72]. Under ambient conditions, the photosynthetic apparatus, consisting of PSII and photosystem I (PSI) on the thylakoid, operates normally and produces large quantities of oxygen that diffuse into the host. Elevated temperature can cause photoinhibition and damage the chloroplast and photosynthetic apparatus that act in concert to start the bleaching cascade [73]. The variation in symbiont photochemistry in our study is clearly a genus-specific response to multiple levels of photodamage triggered by the thermal stress [24], where the D1-D4-D6 symbiont can survive the thermal stress showing unaffected quantum yields of PSII (*F*_v_/*F*_m_ and Δ*F*/*F*_*m*_*'*) and mild adjustment to excitation pressure over PSII (i.e., small Δ*Q*_*m*_) compared with the thermally sensitive C42-C1-C1b-C1c symbiont showing reduced quantum yields and drastic PSII adjustment (i.e., large Δ*Q*_*m*_) (Fig. 2). The difference between the two types of symbionts under thermal stress could also suggest a greater capacity of the D1-D4-D6 symbiont to engage photoprotection or photorepair mechanisms in response to accumulated damage to PSII [23], such as rapid protein turnover [74], upregulated antioxidant activities [75], and adjustment of thylakoid lipid composition [76], which may be one of the reasons that the subsequent translocation of PFC from symbiont to host is unaffected in the PdD holobiont but significantly decreased in PdC (Fig. 3b) [13]. This striking difference is consistent with the results of several other studies of different coral and/or algal species [9, 23], suggesting that corals’ response to thermal stress is highly dependent on the photochemistry of the specific algal symbionts they host.

### Trade-off between coral growth and thermal tolerance

Our measurements of coral skeletal growth indicate that, at control temperature of 26°C, PdC exhibited a 71% higher calcification rate than that in PdD, which correlates with the higher rates of both photosynthetic carbon fixation and the subsequent transfer of PFC from the symbiont to the host (Fig. 3). Similar to our results, Cantin et al. observed that more PFC was translocated to juvenile *A. millepora* harboring type C1 symbiont compared with the juveniles harboring *Durusdinium* symbiont [44]. Similarly, *Cladocopium* was reported to fix and pass significantly more carbon and nitrogen to its coral host (*Isopora palifera)* than *Durusdinium* [77]. These data point to the advantage of hosting *Cladocopium* symbiont for better coral growth under non-elevated temperature condition. This pattern may be coral species-specific as Morikawa et al. found similar results for *P. damicornis* but the opposite in other coral species [78].

However, the superior skeletal growth in PdC was lost after 2 weeks of thermal stress (32°C), whereas the calcification rate in PdD was not affected (Fig. 3a). Similarly, it has been reported that the growth benefit at 26°C for *P. damicornis* hosting metahaplotype C1b-C1c over D1 symbionts was reduced at 30°C [29]. The decreased coral skeletal growth in PdC may be interpreted as thermal stress disturbing photochemistry and carbon fixation of thermally sensitive *Cladocopium* symbionts, which were largely expelled from the coral tissue, and thereby interfering with an important energy supply needed to maintain high calcification rates [79]. Harboring stress-tolerant *Durusdinium* may assist the coral to avoid bleaching under thermal stress but could also incur reduced coral growth (Figs. 1b and 3a). It is suggested that the low rates in carbon fixation and translocation in *Durusdinium* make it act as a “selfish opportunist” [80]. This contradicting outcome of hosting *Cladocopium* versus *Durusdinium* symbiont suggests that the gain in coral resilience to thermal stress contributed by a specific type of symbiont may come at potential trade-offs with other inferior physiological functions intrinsic to that symbiont. From a resource allocation perspective, trade-offs also occur among competing traits representing physiological functions of the host, such as growth, reproduction, and immunity [81]. As a result, many corals enduring thermal challenges show reductions in reproductive capacity [39], translocation of sugars [44], immunity to disease [82], or growth rates [29].

Favoring the proliferation of stress-tolerant but potentially less efficient partners such as *Durusdinium*, hence, appears to be an option that is strongly selected under stressed thermal conditions [65], as seen in many symbiont shuffling/switching cases in the field. For example, surveying *Pocillopora* spp. in the Pacific Panama and a range of species in the Persian/Arabian Gulf and Kenya found corals containing *Durusdinium* were more abundant following episodes of severe, high temperature bleaching [83]. Likewise, symbiont communities changed from *Cladocopium*- to *Durusdinium*-dominated after bleaching in *Acropora millepora*, hence increasing host thermal tolerance by 1–1.5°C [22]. A similar shift was observed in *A. millepora* in the southern Great Barrier Reef following a severe bleaching event [84]. It was also found that *Durusdinium* symbionts in *Montastraea cavernosa* were undetectable by qPCR prior to a bleaching event, but became dominant community members 4–10 months post-bleaching [85]. Last but not least, the prevalence of *Durusdinium* in *P. damicornis* at both the LHT and HH sites (Supplementary Fig. S1c) was likely an adaptive response to increasing anthropogenic and environmental stress accumulated over the years in the southern Hainan Island [45]. Under the context of climate change, the trade-off between coral growth and thermal tolerance appears to be a strategic mechanism in response to increasing environmental perturbations.

### Lineage-specific symbiont dominance, not shuffling, drives distinct coral responses to thermal stress

In this study, by deliberately holding the PdC and PdD corals in the same aquarium to mimic the scenario of symbiont shuffling following a thermal bleaching event, we aimed to elucidate the distinct roles of different algal symbionts in mediating coral response to thermal stress. We found that both the PdC and PdD holobionts maintained their original symbiont dominance during the entire period of thermal stress (Fig. 4a), with only subtle changes of rare (non-dominant) symbiont types at background levels (< 1% of total symbiont population) (Fig. 4b), which is different from previous studies [10–12]. For example, the relative *Durusdinium* to *Cladocopium* abundance (log_10_ [D/C]) in PdC increased from *ca.* 10^−7^ at the start to 10^−4^ at the end of the thermal stress. This 1000-fold increase in *Durusdinium* proportion still renders it at background level, as compared to the > 99% community dominance of *Cladocopium* (Fig. 4a, b). Despite the fluctuations in symbiont profiles or the lack of a single dominant variant, genus-specific samples are similar to each other and grouped, regardless of the treatments, i.e., no stress induced cross-genus symbiont shuffling (Fig. 4c). The lack of symbiont shuffling from *Cladocopium* to *Durusdinium* dominance in PdC may be due to the 2-week thermal stress period that is too short to see any significant symbiont community changes, or the initial extremely low levels of non-dominant symbiont (e.g., *Durusdinium* proportion in PdC was < 0.001% of the symbiont community), which often requires frequent disturbance or sustained warming to allow for the significant proliferation of the rare, opportunistic heat-tolerant genera [80]. In a similar study linking to photodamage, significant symbiont shuffling occurred when the initial *Durusdinium* proportion reached 1% of the symbiont community [10]. On the other hand, the *Durusdinium* community in PdD was rather stable during the long-term aquarium acclimation (i.e., pre-treatment), and the relative *Durusdinium* to *Cladocopium* abundance was even decreased in the simulation experiment under non-elevated temperature condition (Fig. 4). This reversion away from opportunistic symbiont types is probably a result of inferior competitive ability (e.g., lower carbon fixation and translocation) of *Durusdinium* relative to *Cladocopium* during this long periods of “undisturbed” benign conditions [85]. Community reversion back toward a C3-dominated community in bleached corals after 6 months in recovery has been reported [9]. Still, it is worth noting that, even with background shuffling of the non-dominant symbionts, holobiont resilience is, to a larger extent, influenced by the physiology and plasticity of the dominant symbiotic types (Figs. 3 and 4). Therefore, the main objective of characterizing individual symbionts alone in the coral holobiont in our controlled laboratory experiment is to differentiate the roles of *Cladocopium* and *Durusdinium* in mediating coral response to thermal stress. The findings in this study can thus be used as a reference for field observations [22, 85] aiming to elucidate the mechanistic basis of bleaching resistance.

Apart from some probiotic bacteria [1] and endemic symbiont variants [38] that could provide tolerance to thermal stress, symbiont shuffling or switching towards stress-tolerant phenotypes, such as members of *Durusdinium* symbionts, remains a prevalent scheme by which corals adapt to climate change. While such shifts may help reef corals to survive warming conditions [22], non-elevated seawater temperatures during the intervals of bleaching events may favor corals that harbor symbiont types with higher rates of symbiotic carbon fixation and translocation, such as C42 in this study (Fig. 3b, Supplementary Fig. S6). This poses extra concerns on the capacity of coral resilience when facing climate change. As shown in our experiment, while *Durusdinium* proportion is increasing in *P. damicornis* upon thermal stress, the overall *Cladocopium* dominance in PdC cannot offset its thermal susceptibility, manifested by much faster bleaching and reduced calcification and photochemical efficiencies. *Cladocopium* appears to represent the most species-rich and ecologically abundant genus in many Indo-Pacific reef coral communities [2]. Coral bleaching events during the last decade support the notion that the thermal limits of many coral species have already been reached and exceeded [86]. Unless the shift towards thermally tolerant symbiont outcompetes the speed of temperature increase towards the tipping point, the majority of Indo-Pacific reef-building corals dominated by thermally sensitive *Cladocopium* species may be in peril [26].

Last but not least, while thermal tolerance is largely attributed to the type of symbiont in the presented study here, that does not mean that the host genetics could not be playing a significant role. In addition to in-depth exploration of symbiont physiology, there are areas that future studies could spend more time on, especially genomic basis underlining the holobiont response to thermal stress. The application of “omics” approaches (e.g., [87–90]) may provide more clues on how the major coral holobiont constituents including corals, algae, and bacteria interact synergistically to cope with climate change [89], which will undoubtedly facilitate the identification of thermally resistant coral species and inform conservation and restoration efforts.

## Conclusions

**Fig. 5:**
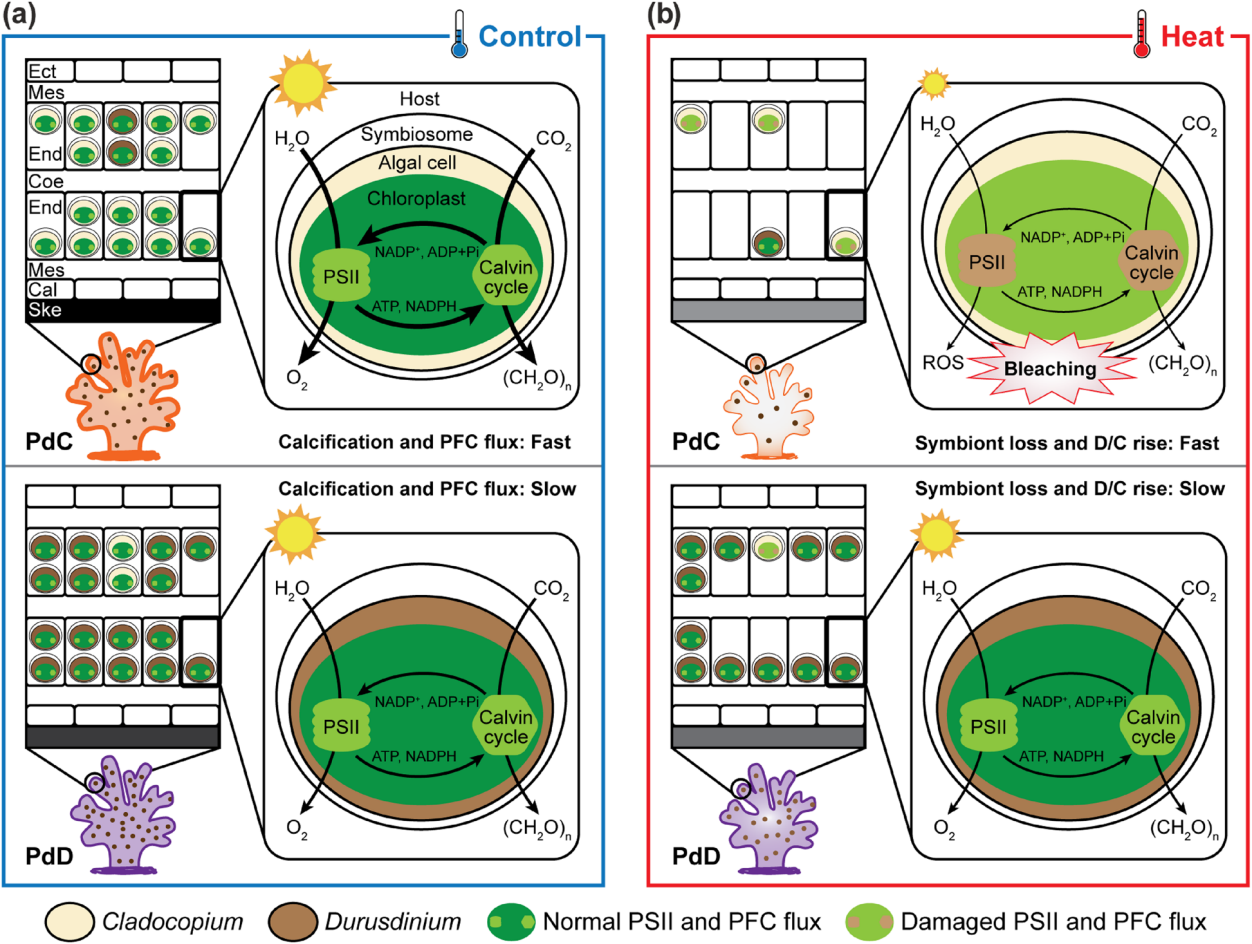
A model explaining how “symbiont dominance” mediates corals’ response to thermal stress. The illustration summarizes the major findings in the present study, depicting how initial symbiont dominance *in hospite*, together with symbiont change in vivo, may affect *P. damicornis* physiology, health, and susceptibility to prolonged heat stress. **a** Under ambient temperature (control), the PdC exhibits higher calcification rate compared to PdD, due to higher rates of photosynthetic carbon fixation and translocation (indicated by large sun image and thick arrows) by *Cladocopium* as compared to *Durusdinium*. **b** Under thermal stress, the rates of photochemistry and carbon fixation are significantly compromised (indicated by small sun image and thin arrows) in *Cladocopium*, possibly due to PSII and chloroplast damage, and a significant loss of the Symbiodiniaceae despite a relatively faster increase in *Durusdinium* proportion in the symbiont community, ultimately leading to reduced calcification rates and coral bleaching in PdC. In contrast, photochemistry and calcification rates are only slightly reduced in the bleaching-tolerant PdD, possibly as a result of dominance of the thermally tolerant *Durusdinium* in the algal community. Coral growth is indicated with the skeleton size and epidermis thickness. Translocation of PFC is indicated with an arrow with the rates indicated by its thickness. Symbiont cells and chloroplasts are color coded to differentiate symbiont type and changes in photochemistry. Ect ectoderm, Mes mesoglea, End endoderm, Cal calcidodermis, Ske skeleton, PFC photosynthetically fixed carbon, D/C *Durusdinium/Cladocopium*, ROS reactive oxygen species

In this study, we aim to investigate the role of Symbiodiniaceae in mediating coral responses to elevated temperatures. Based on our measurement of symbiont density, photochemistry, photosynthetic carbon fixation and translocation, and assessment of coral calcification and bleaching susceptibility, we demonstrate that thermal susceptibility of *P. damicornis* is positively correlated with the physiological performance of the dominant algal symbiont it hosts. When associating with *Durusdinium* symbionts of metahaplotype D1-D4-D6, the holobiont (PdD) was more robust to elevated temperature, maintaining a functional PSII and showing no sign of bleaching. In contrast, *P. damicornis* dominated with *Cladocopium* symbionts of metahaplotype C42-C1-C1b-C1c (PdC) showed disrupted symbiont photochemistry and subsequent translocation of fixed carbon to the host, slowdown of calcification, and bleaching (Fig. 5). However, under ambient temperature condition, both PdC and PdD were healthy and there was no difference in symbiont photochemical efficiency between the two, except that calcification was faster in PdC, seemingly as a result of faster photosynthetic carbon fixation and subsequent translocation to the host. Despite the subtle changes in symbiont relative abundance, we did not observe significant symbiont shuffling during the course of the experiment, as both PdC and PdD maintained their initial symbiont type (> 99% of the symbiont community). The observed thermal susceptibility variation is clearly a trade-off of underlying physiological differences between dominant algal endosymbionts under different temperature regimes. Taken together, these findings shed new light on how dominant symbiont fidelity can affect coral resilience and may aid in the assessment of corals’ ability to persist under global climate change.

### Supplementary Information

**Additional file 1: Fig. S1.** Sampling information and dominant symbionts in *P. damicornis*. **Fig. S2.** Morphology of *P. damicornis*. **Fig. S3.** Neighbor-joining phylogenetic tree reconstructed based on the full-length ITS (ITS1-5.8S-ITS2) sequences amplified in coral nrDNA of *P. damicornis* colonies collected at the LHT and HH fringing reefs. **Fig. S4.** Maximum-parsimony (MP) phylogenetic tree and haplotype network reconstructions of ITS2 sequences amplified from Symbiodiniaceae nrDNA in the selected coral samples collected in HH. **Fig. S5.** Maximum-parsimony (MP) phylogenetic tree and haplotype network reconstructions of ITS2 sequences amplified from Symbiodiniaceae nrDNA in the selected coral samples collected in LHT. **Fig. S6.** Proportion of photosynthetically fixed carbon translocated to host at control and elevated temperatures. **Fig. S7.** Principal component analysis (PCA) of physiological traits mediating overall coral response to heat stress in *P. damicornis*. **Table S1.** The mean, maximum, and minimum sea surface temperatures (SST) in the two sampling sites. **Table S2.** Water quality parameters in the two sampling sites. **Table S3**. Effect of temperature on bleaching rate, Symbiodiniaceae density, photochemical efficiency, and calcification rate in *P. damicornis*. **Table S4.** Generalized linear mixed-effects model comparing the effects of fixed and random factors on physiological traits. **Table S5.** One-way ANOVA assessing the impact of heat stress on physiological traits. **Table S6.** Three-way ANOVA comparing the impacts of temperature, time and symbiont genotype on Symbiodiniaceae density.

## Data Availability

The PCR amplicon sequences were deposited into GenBank (Accession numbers MN394995–395024 for coral ITS sequences and MN394917–394991 and MN876039–876201 for Symbiodiniaceae ITS2 sequences).
