# Supplementary material for: Lineage-specific symbionts mediate differential coral responses to thermal stress
Source: Microbiome. 2023 Sep 26;11:211. doi: 10.1186/s40168-023-01653-4 (PMC10521517; doi:10.1186/s40168-023-01653-4)
Supplement: Supplementary file 2 — Additional file 1: Fig. S1. Sampling information and dominant symbionts in P. damicornis. Fig. S2. Morphology of P. damicornis. Fig. S3. Neighbor-joining phylogenetic tree reconstructed based on the full-length ITS (ITS1-5.8S-ITS2) sequences amplified in coral nrDNA of P. damicornis colonies collected at the LHT and HH fringing reefs. Fig. S4. Maximum-parsimony (MP) phylogenetic tree and haplotype network reconstructions of ITS2 sequences amplified from Symbiodiniaceae nrDNA in the selected coral samples collected in HH. Fig. S5. Maximum-parsimony (MP) phylogenetic tree and haplotype network reconstructions of ITS2 sequences amplified from Symbiodiniaceae nrDNA in the selected coral samples collected in LHT. Fig. S6. Proportion of photosynthetically fixed carbon translocated to host at control and elevated temperatures. Fig. S7. Principal component analysis (PCA) of physiological traits mediating overall coral response to heat stress in P. damicornis. Table S1. The mean, maximum, and minimum sea surface temperatures (SST) in the two sampling sites. Table S2. Water quality parameters in the two sampling sites. Table S3. Effect of temperature on bleaching rate, Symbiodiniaceae density, photochemical efficiency, and calcification rate in P. damicornis. Table S4. Generalized linear mixed-effects model comparing the effects of fixed and random factors on physiological traits. Table S5. One-way ANOVA assessing the impact of heat stress on physiological traits. Table S6. Three-way ANOVA comparing the impacts of temperature, time and symbiont genotype on Symbiodiniaceae density. [file 40168_2023_1653_MOESM1_ESM.docx]

Supplementary Information for

**Lineage-specific symbionts mediate differential coral responses to thermal stress**

Chenying Wang, Xinqing Zheng, Hagit Kvitt, Huaxia Sheng, Danye Sun, Gaofeng Niu, Dan Tchernov, Tuo Shi

Corresponding author: Email: [tuoshi@sdu.edu.cn](mailto:tuoshi@sdu.edu.cn) (T.S.); [zhengxinqing@tio.org.cn](mailto:zhengxinqing@tio.org.cn) (X.Z.); [dtchernov@univ.haifa.ac.il](mailto:dtchernov@univ.haifa.ac.il) (D.T.)

**This file includes:**

Supplementary Figures S1 to S7

Supplementary Tables S1 to S6

**
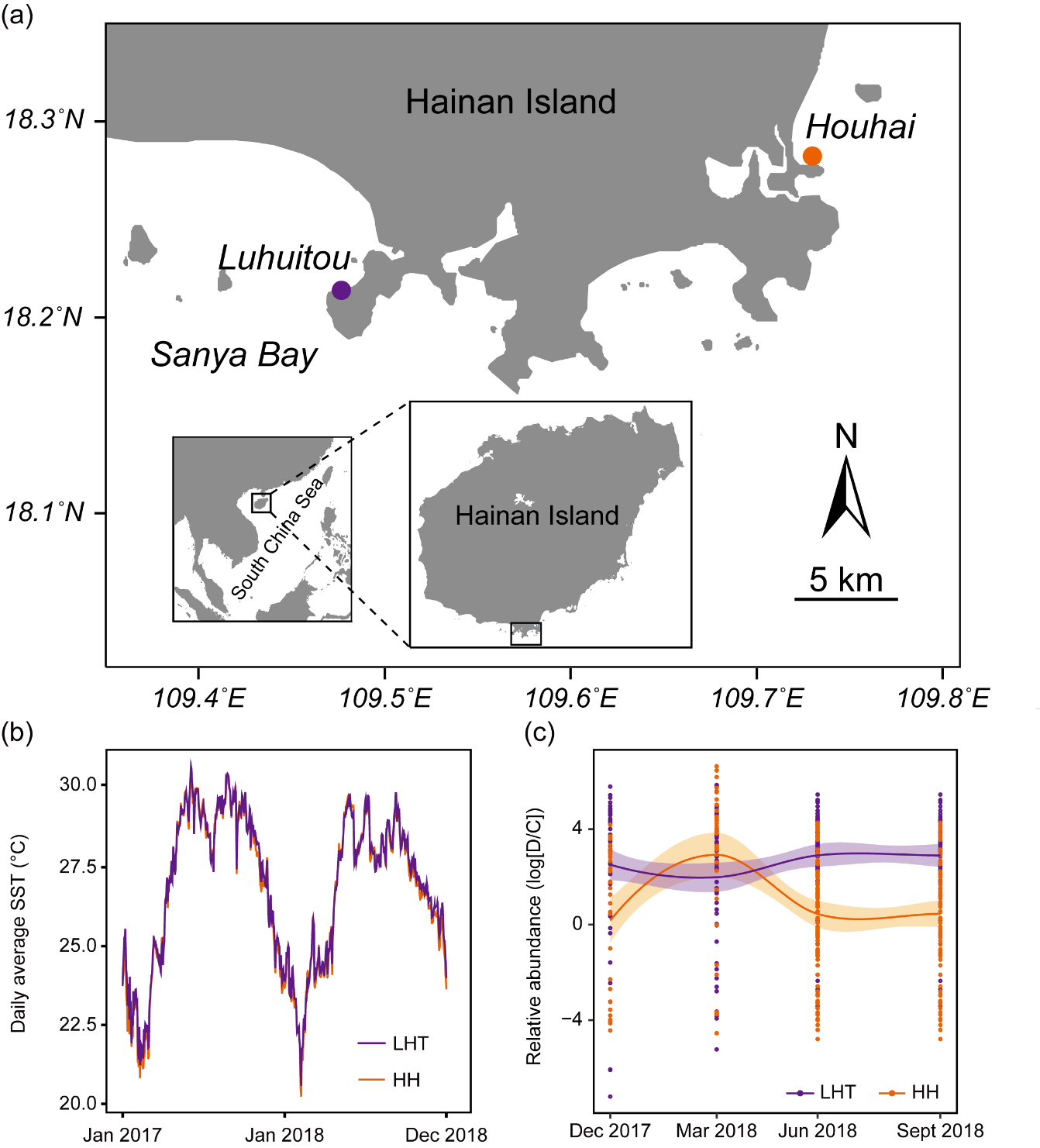
**

**Fig. S1.** **Sampling information and dominant symbionts in *P. damicornis*.** (a) Locations of the two sampling fringing reefs, Luhuitou (LHT; 18°12′7′′N, 109°28′5′′E) and Houhai (HH; 18°16′40′′N, 109°44′3′′E), in southern Hainan Island, China. (b) Daily average sea surface temperatures (SST) in 2017 and 2018 at the two sites. The SST data were obtained from the website <https://psl.noaa.gov/data/gridded/data.noaa.oisst.v2.highres.html>. (c) Relative symbiont abundance expressed as log_10_[D/C] by calculating the *Durusdinium* to *Cladocopium* ratio. Line and shaded area represent logarithmic regression (smoothed data, method = ‘loess’) and 95 % confidence interval, respectively. A total of 538 coral fragments (3–5 cm long) were collected and analyzed during a year-round cross-season survey of symbiont relative abundance in *P. damicornis* at the two sites.


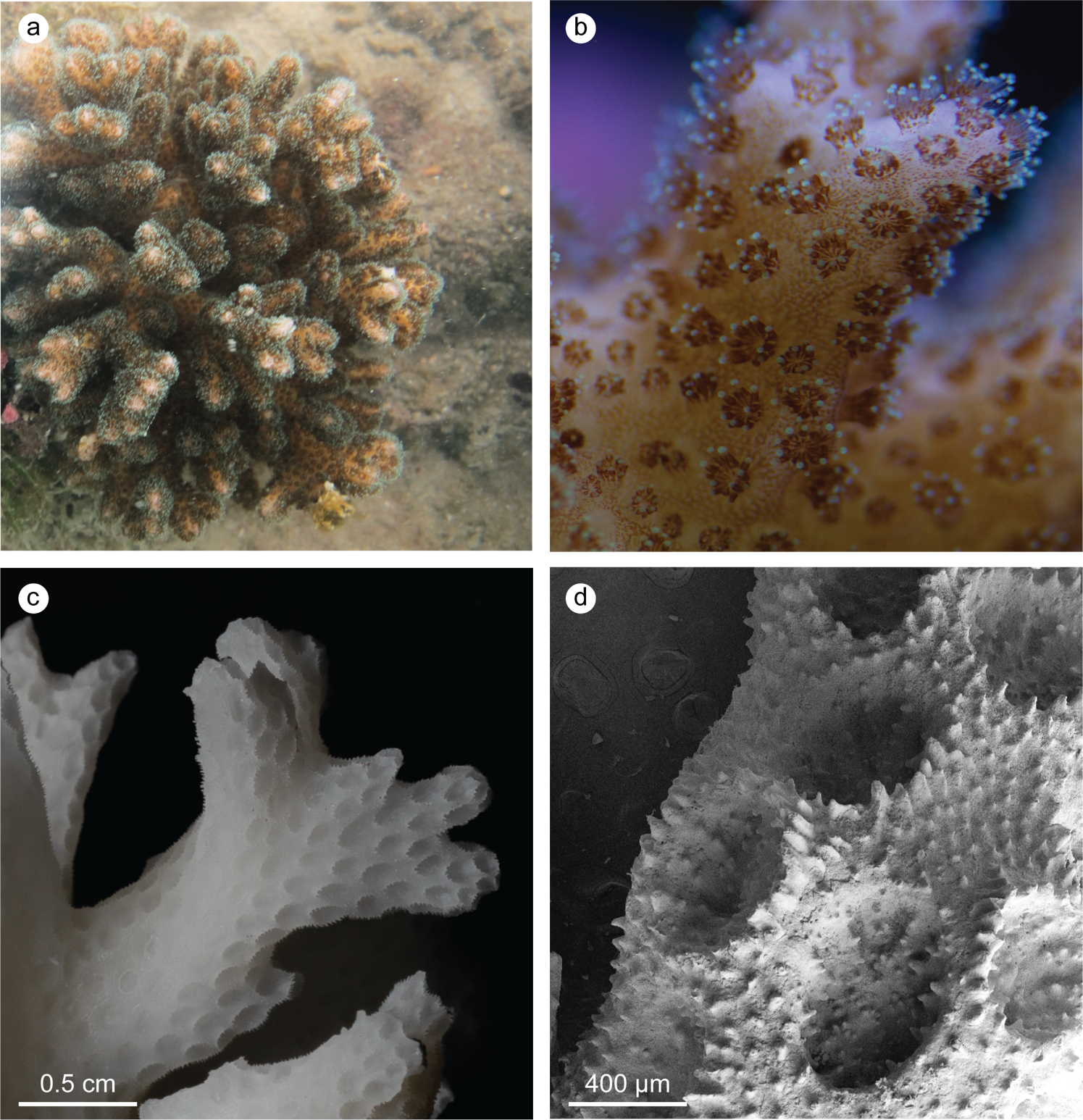


**Fig. S2.** **Morphology of *P. damicornis***. (a) An isolated colony that appears brownish due to the Symbiodiniaceae it contains. (b) Closeup of polyps showing tentacles and pigmented Symbiodiniaceae surrounding the oral disc. (c) Closeup of the skeleton showing array of calices. (d) Scanning electron microscopic close-up of corallites showing detailed structures of skeletal spines overlaying the coenosteum and calice.


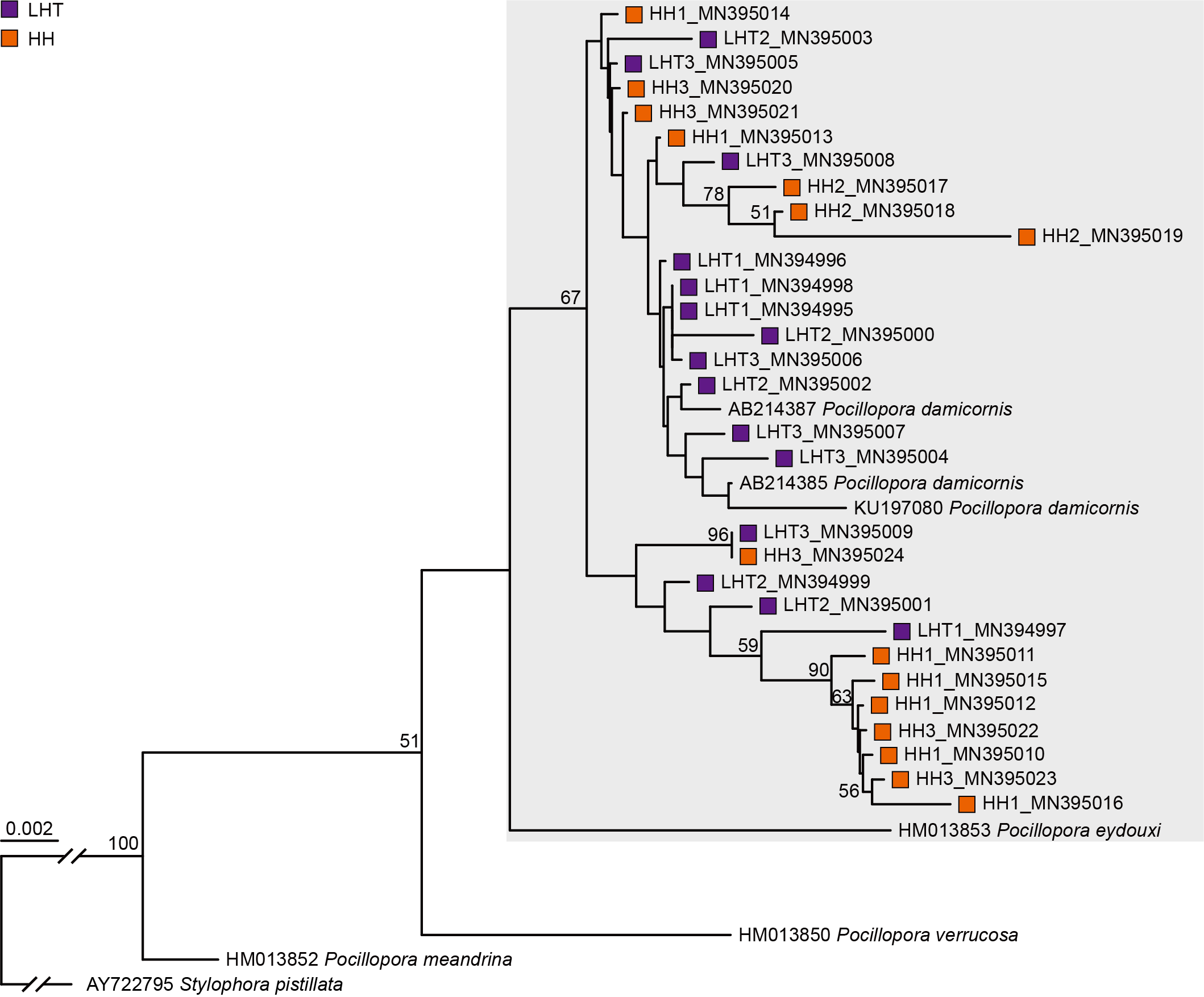


**Fig. S3.** **Neighbor-joining phylogenetic tree reconstructed based on the full-length ITS (ITS1-5.8S-ITS2) sequences amplified in coral nrDNA of *P. damicornis* colonies collected at the LHT and HH fringing reefs.** Up to six clones of the PCR amplicons were sequenced for each of the three colonies collected at each site, indicated by location and replicate number, i.e., LHT1–3 and HH1–3 followed by GenBank accession. Reference sequences of different Pocilloporid corals were included to facilitate species identification of the collected coral samples. The phylogeny was rooted with sequences of *Stylophora pistillata* as the outgroup. Branching support over 50 % out of 1,000 bootstrap replicates is marked. Tree scale represents number of substitutions per site.

**
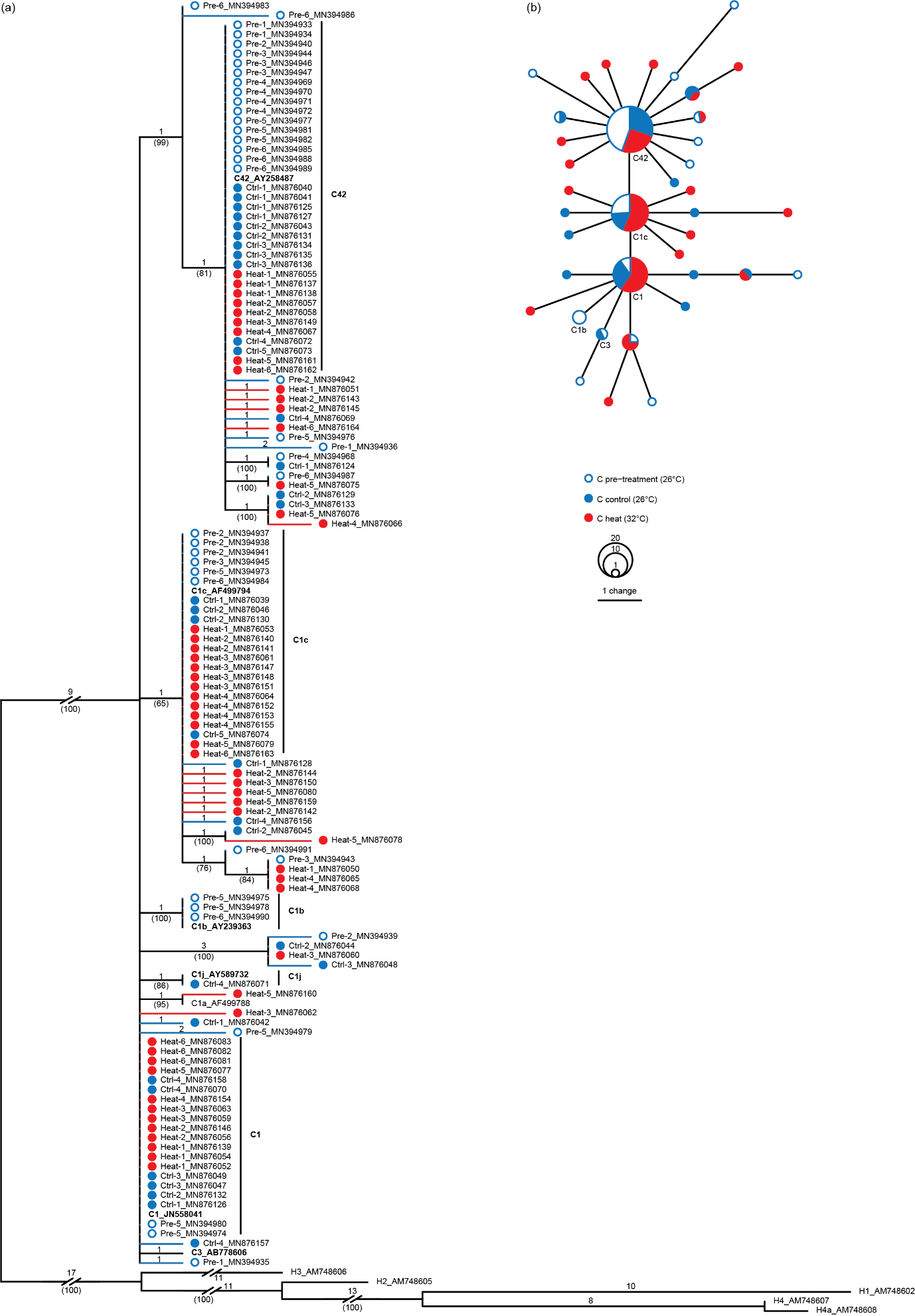
**

**Fig. S4. Maximum-parsimony (MP) phylogenetic tree and haplotype network reconstructions of ITS2 sequences amplified from Symbiodiniaceae nrDNA in the selected coral samples collected in HH**. (a) MP tree and (b) haplotype network of all cloned sequences from *P. damicornis* in HH hosting C42 and closely related symbionts in *Cladocopium*. Hexaplicate (n = 6) coral fragments harvested prior to the experiment (indicated as Pre-1–6 for the pre-treatment), as well as those harvested at the end of the thermal stress manipulation (indicated as Ctrl-1–6 for the control and Heat-1–6 for the heated temperature). The *Cladocopium* phylogeny was rooted with selected reference sequences in *Halluxium* (formerly clade H) as the outgroup. Numerals above each branch indicate number of informative base pair differences. Numbers in parentheses are branching support values (percentage) out of 200 bootstrap replicates. The size of circles in haplotype network represents the numbers of clones with identical sequences at each node.

**
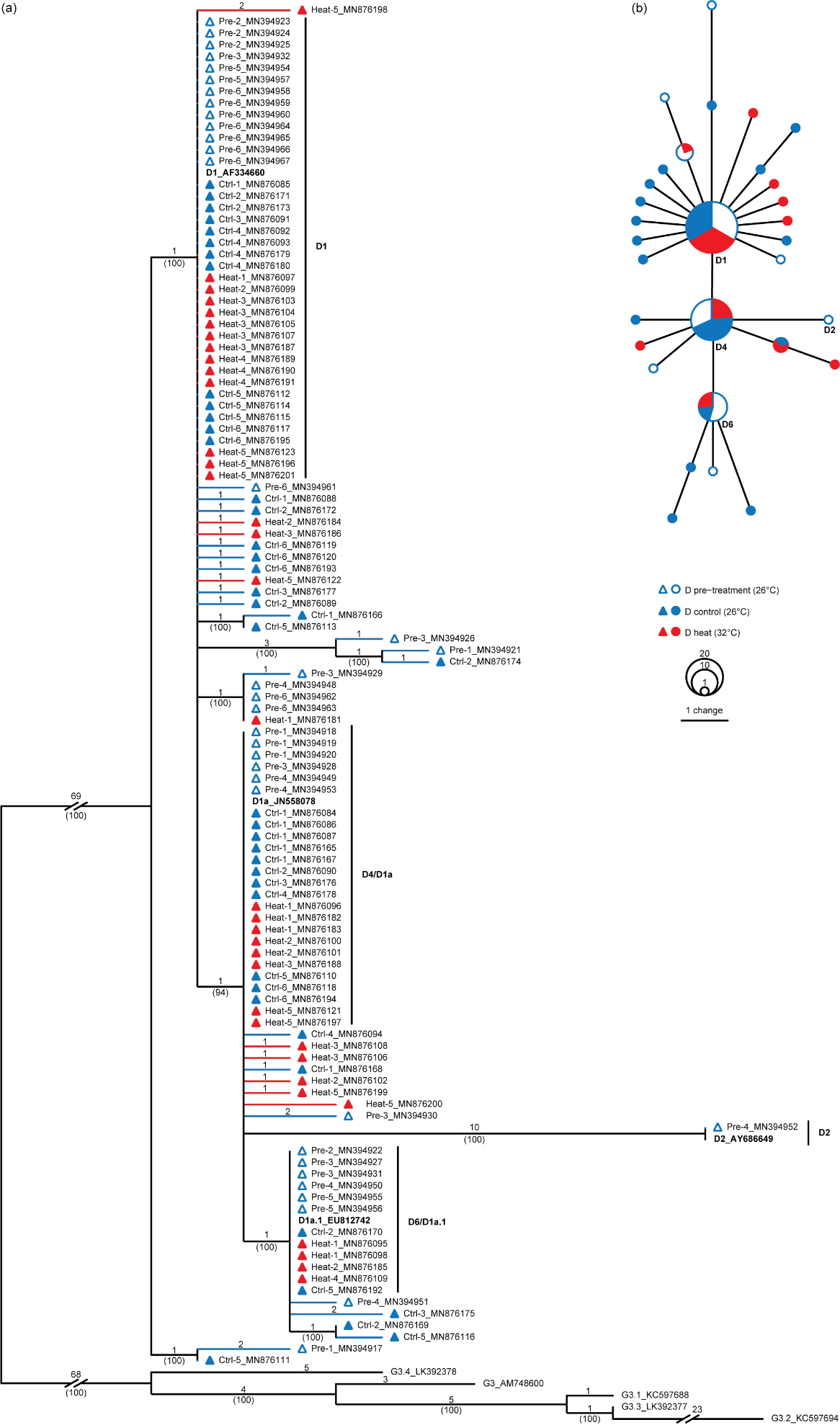
**

**Fig. S5. Maximum-parsimony (MP) phylogenetic tree and haplotype network reconstructions of ITS2 sequences amplified from Symbiodiniaceae nrDNA in the selected coral samples collected in LHT**. (a) MP tree and (b) haplotype network of *P. damicornis* in LHT harboring D1 and closely related symbionts in *Durusdinium*. Hexaplicate (n = 6) coral fragments harvested prior to the experiment (indicated as Pre-1–6 for the pre-treatment), as well as those harvested at the end of the thermal stress manipulation (indicated as Ctrl-1–6 for the control and Heat-1–6 for the heated temperature). The *Durusdinium* phylogeny was rooted with selected reference sequences in *Gerakladium* (formerly clade G) as the outgroup. Numerals above each branch indicate number of informative base pair differences. Numbers in parentheses are branching support values (percentage) out of 200 bootstrap replicates. The size of circles in haplotype network represents the numbers of clones with identical sequences at each node.


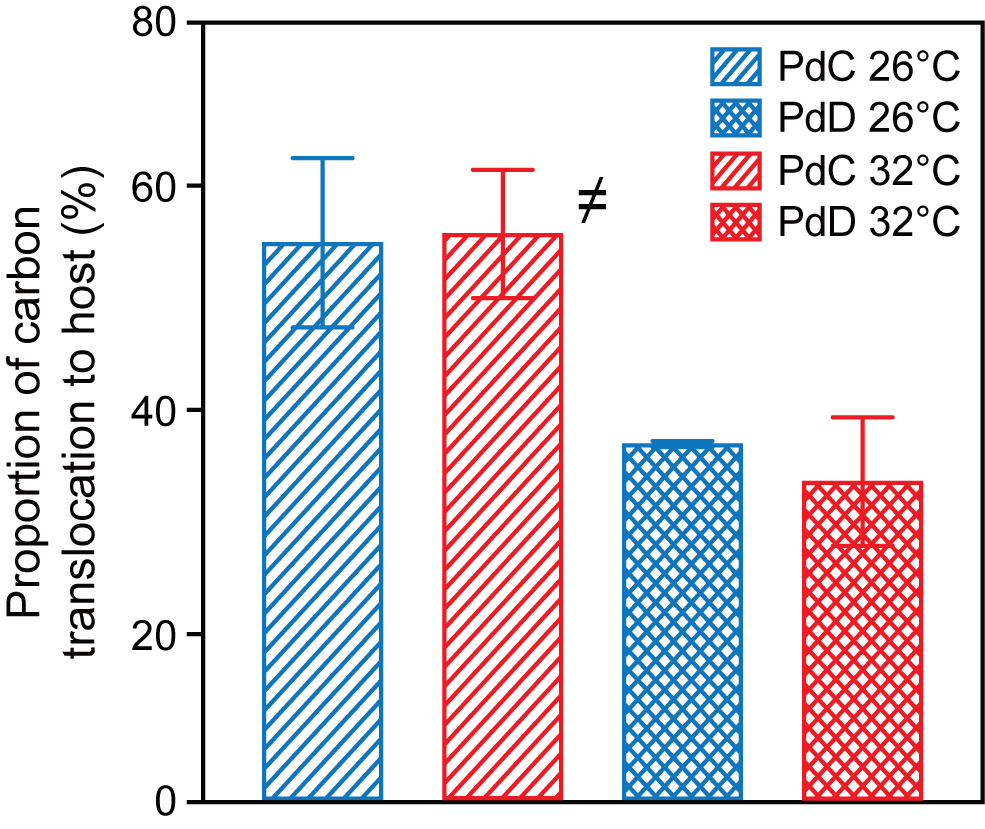


**Fig. S6. Proportion of photosynthetically fixed carbon translocated to host at control and elevated temperatures**. Stable isotopic tracing was performed after 14 d of heat stress. The inequality sign (≠) indicates significant differences between corals harboring *Cladocopium* or *Durusdinium* at both the control and elevated temperatures (Tukey’s *post hoc* test, *p* < 0.05).


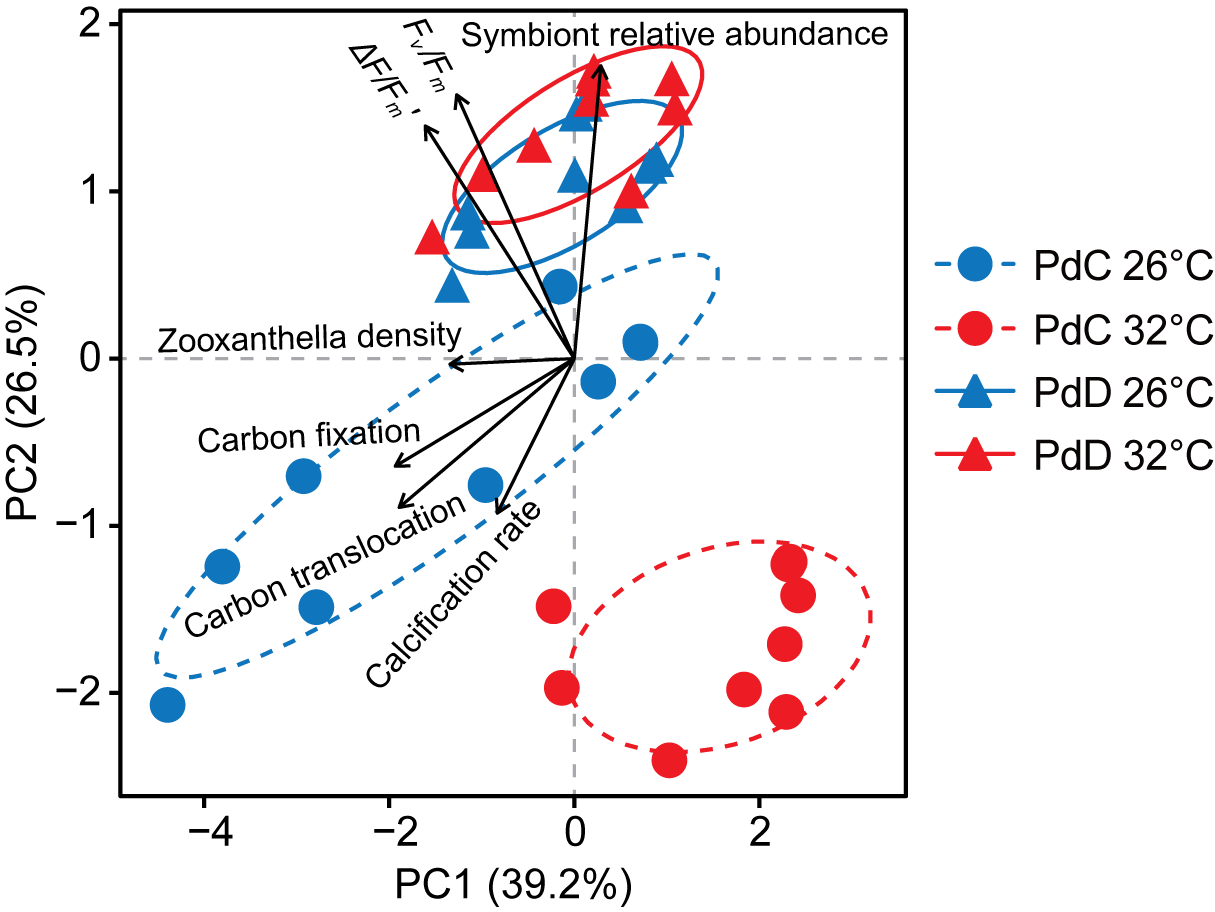


**Fig. S7. Principal component analysis (PCA) of physiological traits mediating overall coral response to heat stress in *P. damicornis***. Almost all of the physiological traits (expect symbiont relative abundance) were correlated and accounted for 39.2 % variation along PC1, whereas *F_v_/F_m_*, Δ*F/F_m_'*, and symbiont relative abundance also separated data and explained 21 % variation along PC2. These physiological traits were therefore significant in driving separation of samples among the four treatments. Each treatment is grouped, with the ellipse representing 80 % confidence.

**Table S1.** **The mean, maximum, and minimum sea surface temperatures (SST) in the two sampling sites.**

|  | LHT | | | HH | | |
| --- | --- | --- | --- | --- | --- | --- |
| Factor | Mean | Max | Min | Mean | Max | Min |
| SST | 26.8 | 30.7 | 20.6 | 26.7 | 30.5 | 20.2 |

The data were derived from daily averages in 2017 and 2018 downloaded from the website https://psl.noaa.gov/data/gridded/data.noaa.oisst.v2.highres.html.

**Table S2. Water quality parameters in the two sampling sites**.

| Time | Location | PO_4_^3+^-P  (μmol/L) | NO_2_^–^-N  (μ mol/L) | NO_3_^–^-N  (μmol/L) | SiO_3_^2–^-Si  (μmol/L) | NH_4_^+^-N  (μmol/L) |
| --- | --- | --- | --- | --- | --- | --- |
| Dec 2017 | LHT | 0.18 ± 0.00 ^a^ | 0.11 ± 0.00 ^a^ | 0.02 ± 0.02 ^a^ | 3.15 ± 0.26 ^a^ | 0.46 ± 0.06 ^a^ |
|  | HH | 0.16 ± 0.01 ^a^ | 0.13 ± 0.01 ^a^ | 0 ^a^ | 2.92 ± 0.07 ^a^ | 0.43 ± 0.05 ^a^ |
| Mar 2018 | LHT | 0.00 ± 0.00 ^a^ | 0.08 ± 0.00 ^a^ | 0 ^a^ | 7.51 ± 0.39 ^a^ | 0 ^a^ |
|  | HH | 0.00 ± 0.00 ^a^ | 0.07 ± 0.01 ^a^ | 0 ^a^ | 7.06 ± 0.19 ^a^ | 0 ^a^ |
| Jun 2018 | LHT | 0.18 ± 0.02 ^a^ | 0.13 ± 0.02 ^a^ | 0.06 ± 0.02 ^a^ | 6.40 ± 0.15 ^a^ | 0.73 ± 0.25 ^a^ |
|  | HH | 0.20 ± 0.01 ^a^ | 0.11 ± 0.01 ^a^ | 0.04 ± 0.02 ^a^ | 5.92 ± 0.34 ^a^ | 0.64 ± 0.07 ^a^ |
| Sept 2018 | LHT | 0.15 ± 0.00 ^a^ | 0.10 ± 0.01 ^a^ | 0 ^a^ | 3.19 ± 0.07 ^a^ | 0.53 ± 0.18 ^a^ |
|  | HH | 0.14 ± 0.01 ^a^ | 0.11 ± 0.01 ^a^ | 0.01 ± 0.01 ^a^ | 3.28 ± 0.18 ^a^ | 0.50 ± 0.17 |

Values are expressed as the mean ± standard error (n = 4). For each parameter measured, different superscript letters, if any, represent statistically significant difference between the two locations at designated months (*p* < 0.05).

**Table S3**. **Effect of temperature on bleaching rate, Symbiodiniaceae density, photochemical efficiency, and calcification rate in *P. damicornis*.**

| Time | Treatment | Bleaching rate (%) | Symbiodiniaceae density (cells cm^–2^) | Δ*F*/*F_m_*′ | *F_v_/F_m_* | Calcification rate (g CaCO_3_ cm^–2^ d^–1^) | Photosynthetic carbon fixation | Carbon Translocation to host |
| --- | --- | --- | --- | --- | --- | --- | --- | --- |
| 0 days | PdC 26℃ | 0 | 3118013 ± 92321 | 0.616 ± 0.0061 | 0.666 ± 0.0096 | 0.00794 ± 0.00173 | _ | _ |
|  | PdC 32℃ | 0 | 2788373 ± 431502 | 0.631 ± 0.0110 | 0.663 ± 0.0035 | 0.00755 ± 0.00129 | _ | _ |
|  | PdD 26℃ | 0 | 6282968 ± 287123 | 0.622 ± 0.0076 | 0.659 ± 0.0144 | 0.00293 ± 0.00062 | _ | _ |
|  | PdD 32℃ | 0 | 5916754 ± 517257 | 0.615 ± 0.0074 | 0.624 ± 0.0346 | 0.00250 ± 0.00092 | _ | _ |
| 7 days | PdC 26℃ | 0 | 2980278 ± 46602 | 0.551 ± 0.0149 | 0.659 ± 0.0056 | 0.00887 ± 0.00205 | _ | _ |
|  | PdC 32℃ | 26.7 ± 3.33 | 474102 ± 235325 | 0.341 ± 0.0220 | 0.520 ± 0.0114 | 0.00524 ± 0.00136 | _ | _ |
|  | PdD 26℃ | 0 | 5648214 ± 346796 | 0.544 ± 0.0070 | 0.649 ± 0.0079 | 0.00251 ± 0.00092 | _ | _ |
|  | PdD 32℃ | 0 | 5713514 ± 373287 | 0.525 ± 0.0101 | 0.623 ± 0.0135 | 0.00246 ± 0.00047 | _ | _ |
| 14 days | PdC 26℃ | 0 | 2950078 ± 252670 | 0.506 ± 0.0194 | 0.623 ± 0.0156 | 0.00765 ± 0.00136 | 344.6 ± 36.37 | 200.61 ± 39.22 |
|  | PdC 32℃ | 83.3 ± 3.33 | 63875 ± 47046 | 0.072 ± 0.0147 | 0.144 ± 0.0239 | 0.00361 ± 0.00110 | 107.68 ± 37.35 | 68.21 ± 26.83 |
|  | PdD 26℃ | 0 | 5287779 ± 149200 | 0.511 ± 0.0237 | 0.656 ± 0.0180 | 0.00265 ± 0.00068 | 204.19 ± 13.25 | 74.84 ± 4.94 |
|  | PdD 32℃ | 0 | 4085175 ± 838304 | 0.457 ± 0.0145 | 0.600 ± 0.0214 | 0.00231 ± 0.00094 | 207.43 ± 54.97 | 63.39 ± 17.34 |

Values are expressed as mean ± standard error.

**Table S4. Generalized linear mixed-effects model comparing the effects of fixed and random factors on physiological traits.**

| Dependent variable | Factors | *F* | *p* |
| --- | --- | --- | --- |
| Symbiodiniaceae density (cells cm^–2^) | Temperature | 10.346 | **0.003** |
|  | Time | 3.922 | **0.030** |
|  | Genotype | 21.217 | **0.000** |
|  | Tank*Colony | _ | 0.325 |
| Δ*F*/*F_m_*′ | Temperature | 35.157 | **0.000** |
|  | Time | 36.327 | **0.000** |
|  | Genotype | 16.060 | **0.000** |
|  | Tank*Colony | _ | 0.073 |
| *F_v_/F_m_* | Temperature | 19.387 | **0.000** |
|  | Time | 9.614 | **0.000** |
|  | Genotype | 11.284 | **0.001** |
|  | Tank*Colony | _ | 0.090 |
| Calcification rate (g CaCO_3_ cm^–2^ d^–1^) | Temperature | 4.130 | **0.045** |
|  | Time | 0.660 | 0.519 |
|  | Genotype | 38.260 | **0.000** |
|  | Tank*Colony | _ | 0.997 |
| Symbiont relative abundance | Temperature | 18.006 | **0.000** |
|  | Time | 6.639 | **0.002** |
|  | Genotype | 1180.470 | **0.000** |
|  | Tank*Colony | _ | 0.148 |

Significant differences (*p* < 0.05) are highlighted in bold. *F*: *F*-ratio; *p*: *p*-value.

**Table S5. One-way ANOVA assessing the impact of heat stress on physiological traits**.

| Dependent variable | Time | Treatment | SS | *df* | MS | *F* | *p* |
| --- | --- | --- | --- | --- | --- | --- | --- |
| Bleaching rate (%) | PdC | 0 day | 0 | 1 | 0 | _ | _ |
|  |  | 7 day | 1067 | 1 | 1067 | 64 | **0.0013** |
|  |  | 14 day | 10417 | 1 | 10417 | 625 | **1.52e-05** |
|  | PdD | 0 day | 0 | 1 | 0 | _ | _ |
|  |  | 7 day | 0 | 1 | 0 | _ | _ |
|  |  | 14 day | 0 | 1 | 0 | _ | _ |
| Symbiodiniaceae density (cells cm^–2^) | PdC | 0 day | 1.630e+11 | 1 | 1.630e+11 | 0.558 | 0.4970 |
|  |  | 7 day | 9.421e+12 | 1 | 9.421e+12 | 109.1 | **0.0005** |
|  |  | 14 day | 1.250e+13 | 1 | 1.250e+13 | 126.1 | **0.0004** |
|  | PdD | 0 day | 2.012e+11 | 1 | 2.012e+11 | 0.383 | 0.5690 |
|  |  | 7 day | 6.396e+09 | 1 | 6.396e+09 | 0.016 | 0.9040 |
|  |  | 14 day | 2.169e+12 | 1 | 2.169e+12 | 1.995 | 0.2310 |
| Δ*F*/*F_m_*′ | PdC | 0 day | _ | 1 | _ | _ | 0.2360 |
|  |  | 7 day | 0.4057 | 1 | 0.4057 | 63.41 | **2.28e-09** |
|  |  | 14 day | 0.4705 | 1 | 0.4705 | 318.1 | **1e-07** |
|  | PdD | 0 day | 0.0003 | 1 | 0.0003 | 0.419 | 0.5240 |
|  |  | 7 day | _ | 1 | _ | _ | 0.0535 |
|  |  | 14 day | 0.0105 | 1 | 0.0105 | 2.919 | 0.1110 |
| *F_v_/F_m_* | PdC | 0 day | 0.00004 | 1 | 0.00005 | 0.093 | 0.7640 |
|  |  | 7 day | 0.1331 | 1 | 0.1331 | 140.2 | **5.65e-12** |
|  |  | 14 day | 0.4819 | 1 | 0.4819 | 282.5 | **1.59e-07** |
|  | PdD | 0 day | _ | 1 | _ | _ | 0.0991 |
|  |  | 7 day | _ | 1 | 0.0053 | _ | 0.0669 |
|  |  | 14 day | 0.012 | 1 | 0.0118 | 3.243 | 0.0933 |
| Calcification rate (g CaCO_3_ cm^–2^ d^–1^) | PdC | 0 day | _ | 1 | _ | _ | 0.7624 |
|  |  | 7 day | _ | 1 | _ | _ | **0.0413** |
|  |  | 14 day | _ | 1 | _ | _ | **0.0191** |
|  | PdD | 0 day | _ | 1 | _ | _ | 0.1509 |
|  |  | 7 day | _ | 1 | _ | _ | 0.1988 |
|  |  | 14 day | _ | 1 | _ | _ | 0.3258 |

Significant differences (*p* < 0.05) between control and elevated temperatures are highlighted in bold. SS: type III sum of squares; *df*: degree of freedom; MS: mean square; *F*: *F*-ratio; *p*: *p*-value.

**Table S6. Three-way ANOVA comparing the impacts of temperature, time and symbiont genotype on Symbiodiniaceae density.**

| Symbiodiniaceae density (cells cm^–2^) | SS | *df* | MS | *F* | *p* |
| --- | --- | --- | --- | --- | --- |
| Temperature | 1.305e+13 | 1 | 1.305e+13 | 31.585 | **0.000** |
| Time | 1.236e+13 | 2 | 6.179e+12 | 14.953 | **0.000** |
| Genotype | 1.057e+14 | 1 | 1.057e+14 | 255.726 | **0.000** |
| Temperature*Time | 4.318e+12 | 2 | 2.159e+12 | 5.225 | **0.013** |
| Temperature*Genotype | 4.449e+12 | 1 | 4.449e+12 | 10.766 | **0.003** |
| Time*Genotype | 1.252e+12 | 2 | 6.258e+11 | 1.514 | 0.240 |
| Temperature*Time*Genotype | 2.637e+12 | 2 | 1.319e+12 | 3.191 | 0.059 |

Significant differences (*p* < 0.05) are highlighted in bold. SS: type III sum of squares; *df*: degree of freedom; MS: mean square; *F*: *F*-ratio; *p*: *p*-value.
